# Engineering *Corynebacterium glutamicum* for the production of 2,3-butanediol

**DOI:** 10.1186/s12934-015-0362-x

**Published:** 2015-10-29

**Authors:** Dušica Radoš, Ana Lúcia Carvalho, Stefan Wieschalka, Ana Rute Neves, Bastian Blombach, Bernhard J. Eikmanns, Helena Santos

**Affiliations:** Instituto de Tecnologia Química e Biológica, Universidade Nova de Lisboa, Av. da República-EAN, 2780-157 Oeiras, Portugal; Institute of Microbiology and Biotechnology, University of Ulm, 89069 Ulm, Germany; Institute for Biochemical Engineering, University of Stuttgart, 70569 Stuttgart, Germany; Lisbon Academy of Sciences, R. Academia das Ciências 19, 1249 Lisbon, Portugal; CED-Discovery, Chr Hansen A/S, 10-12 Bøge Alle, 2970 Hørsholm, Denmark; Gut Health and Food Safety Programme, Institute of Food Research, Norwich Research Park, Norwich, UK; Rentschler Biotechnologie GmbH, Erwin-Rentschler-Straße, 21, 88471 Laupheim, Germany

**Keywords:** 2,3-butanediol, *Corynebacterium glutamicum*, Metabolic engineering, Pyruvate node, *Lactococcus lactis*

## Abstract

**Background:**

2,3-Butanediol is an important bulk chemical with a wide range of applications. In bacteria, this metabolite is synthesised from pyruvate via a three-step pathway involving α-acetolactate synthase, α-acetolactate decarboxylase and 2,3-butanediol dehydrogenase. Thus far, the best producers of 2,3-butanediol are pathogenic strains, hence, the development of more suitable organisms for industrial scale fermentation is needed. Herein, 2,3-butanediol production was engineered in the Generally Regarded As Safe (GRAS) organism *Corynebacterium glutamicum*. A two-stage fermentation process was implemented: first, cells were grown aerobically on acetate; in the subsequent production stage cells were used to convert glucose into 2,3-butanediol under non-growing and oxygen-limiting conditions.

**Results:**

A gene cluster, encoding the 2,3-butanediol biosynthetic pathway of *Lactococcus lactis*, was assembled and expressed in background strains, *C. glutamicum* Δ*ldhA*, *C. glutamicum* Δ*aceE*Δ*pqo*Δ*ldhA* and *C. glutamicum* Δ*aceE*Δ*pqo*Δ*ldhA*Δ*mdh*, tailored to minimize pyruvate-consuming reactions, i.e., to prevent carbon loss in lactic, acetic and succinic acids. Producer strains were characterized in terms of activity of the relevant enzymes in the 2,3-butanediol forming pathway, growth, and production of 2,3-butanediol under oxygen-limited conditions. Productivity was maximized by manipulating the aeration rate in the production phase. The final strain, *C. glutamicum* Δ*aceE*Δ*pqo*Δ*ldhA*Δ*mdh*(pEKEx2-*als,aldB,*P_tuf_*butA*), under optimized conditions produced 2,3-butanediol with a 0.66 mol mol^−1^ yield on glucose, an overall productivity of 0.2 g L^−1^ h^−1^ and a titer of 6.3 g L^−1^.

**Conclusions:**

We have successfully developed *C. glutamicum* into an efficient cell factory for 2,3-butanediol production. The use of the engineered strains as a basis for production of acetoin, a widespread food flavour, is proposed.

**Electronic supplementary material:**

The online version of this article (doi:10.1186/s12934-015-0362-x) contains supplementary material, which is available to authorized users.

## Background

Concern over exhaustion of fossil fuel resources, emission of CO_2_ linked with petroleum-derived products, and accumulation of non-degradable synthetic polymers urged the development of environmentally friendly processes for production of chemicals. A solution offered by the rising field of white biotechnology is to produce chemical building blocks by microbial fermentation of sugars derived from renewable biomass [1].

2,3-Butanediol (2,3-BD) is an important chemical used in the production of plasticizers and fumigants, as an antifreeze agent, a fuel and octane booster, among other applications [2]. The range is still expanding and the market size is expected to reach 74 kilo tons by 2018 [3]. Significantly, the 2,3-BD derivative, 1,3-butanediene, can be used in synthetic rubber production, while 2-butanone (methyl ethyl ketone) is a fuel additive and solvent for resins and lacquers. Additionally, ester-derivatives are used in the pharmaceutical and cosmetics industries [2]. The world annual market for 2,3-BD derivatives is estimated at around $43 billion [4].

**Fig. 1:**
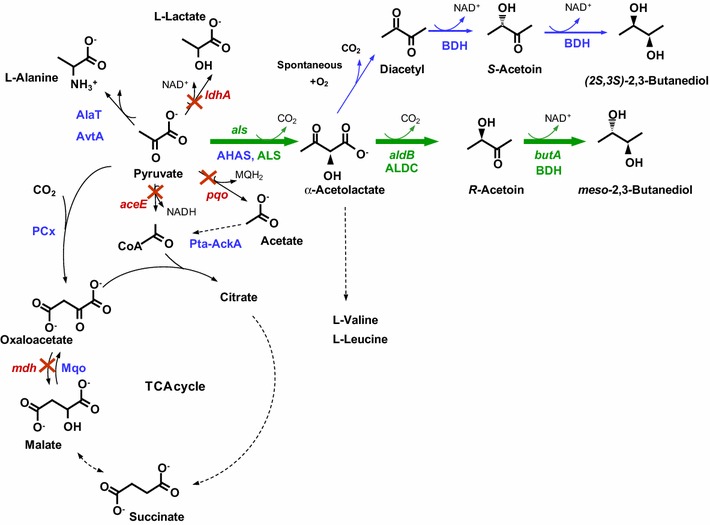
A scheme depicting major pyruvate consuming reactions, including the proposed pathway for synthesis of optically active 2,3-BD in *C. glutamicum* and the strategy for engineering *meso*-2,3-BD synthesis. *L. lactis* genes of the 2,3-BD biosynthetic pathway (*green*) were introduced into *C. glutamicum*. Endogenous genes of interest are presented in *blue*; the *red cross marks* indicate genes that were inactivated in host strains to prevent the production of lactic acid (*ldhA*) and acetic acid (*aceE* and *pqo*); suppression of succinic acid was attempted by deletion of *mdh*. *mdh*, malate dehydrogenase gene; *mqo*, malate:quinone oxidoreductase gene; ALS, α-acetolactate synthase (encoded by *als*); ALDC, α-acetolactate decarboxylase (encoded by *aldB*); BDH, butanediol dehydrogenase (encoded by *butA*), AHAS, acetohydroxyacid synthase; LDH, lactate dehydrogenase (encoded by *ldhA*); PQO, pyruvate:quinone oxidoreductase (encoded by *pqo*); AlaT, alanine-glutamate transaminase; AvtA, alanine-valine transaminase; AceE, E1-subunit of the pyruvate dehydrogenase complex (encoded by *aceE*); Pta-Ack, phosphotransacetylase and acetate kinase; PCx, pyruvate carboxylase

2,3-BD has three stereoisomeric forms: the enantiomers (*2S,3S*)-2,3-BD and (*2R,3R*)-2,3-BD, and the optically inactive form (*2R,3S*)-2,3-BD (*meso*-2,3-BD). 2,3-BD is an end-product of the metabolism of many bacteria, synthesized from pyruvate via a three step pathway (Fig. 1). The first reaction involves the condensation of two pyruvate molecules into α-acetolactate, which is catalyzed by α-acetolactate synthase (ALS, EC 2.2.1.6), the enzyme committed to pyruvate catabolism. Alternatively, as in *Corynebacterium glutamicum*, α-acetolactate can be synthesized by the action of acetohydroxyacid synthase (AHAS, EC 2.2.1.6, encoded by *ilvBN*), and used as precursor for the synthesis of branched chain amino acids (l-valine, l-leucine and l-isoleucine). In the catabolic route, α-acetolactate is decarboxylated by α-acetolactate decarboxylase (ALDC, EC 4.1.1.5, encoded by *aldB*) to yield *R*-acetoin. In the presence of oxygen, α-acetolactate can undergo spontaneous decarboxylation to form diacetyl, which is subsequently reduced to *R*- or *S*-acetoin by diacetyl reductases (EC 1.1.1.303; EC 1.1.1.304). Acetoin is finally reduced to 2,3-BD by butanediol dehydrogenases (BDH, EC 1.1.1.76; EC 1.1.1.4). In some cases, such as *C. glutamicum* or *L. lactis*, BDH is promiscuous and recognizes as substrates diacetyl as well as acetoin, thus diacetyl can be reduced to acetoin via this activity [5, 6].

Thus far, the best producers of 2,3-BD are pathogenic strains, such as *Klebsiella pneumoniae*, *Klebsiella oxytoca*, *Enterobacter aerogenes*, and *Serratia marcescens* [7–10], hence, a considerable research effort has been directed to the development of more suitable organisms for industrial scale fermentation. *Lactococcus lactis*, *Bacillus subtilis*, *Paenibacillus polymyxa*, *Saccharomyces cerevisiae* and *Escherichia coli* have been used for this purpose, but the low production efficiency or complex nutritional requirements of the engineered strains make the overall process unsatisfactory [11–15]. Therefore, the search goes on for more appropriate host strains and more efficient fermentation processes. A few years ago, some of us engineered *L. lactis* for the production of 2,3-BD by overexpression of two endogenous genes (*als*, *butA*) [11]; theoretical product yields were reached, but the fastidious nutritional requisites of this bacterium precludes its utilization as an industrial producer of bulk chemicals.

In this work, we selected *Corynebacterium glutamicum* as an industrially established host strain and aimed to develop an efficient 2,3-BD producer. *C. glutamicum* is an important industrial microorganism, widely known for the million-ton scale production of l-glutamate and l-lysine [16]. Therefore, the existing knowledge of the industrial fermentation of this bacterium could be exploited for the production of bulk chemicals, such as 2,3-BD. In addition, *C. glutamicum* is a facultative anaerobic organism with GRAS (Generally Regarded As Safe) status, robust (osmotolerant, phageresistant, organic solvent tolerant), extensively studied with respect to metabolism and regulation, and for which a comprehensive toolbox for genetic manipulation has been developed [17–19]. Considerable research effort has been invested in metabolic engineering of *C. glutamicum,* resulting in the construction of producer strains for a broad spectrum of compounds, such as biofuels (isobutanol and ethanol), polymer precursors (diaminopentane, cadaverine and putrescine), sugar alcohols (xylitol) and organic acids [20–29]. As a result of this research endeavour, platform strains of *C. glutamicum* have been established. More specifically, strains deficient in l-lactate dehydrogenase (LDH, encoded by *ldhA*) have been proven to be a useful starting point for strain development since deletion of the *ldhA* gene eliminates l-lactate formation, concomitantly increasing NADH availability [27, 29]. In addition, strains deficient in the *aceE*-encoded E1 subunit of the pyruvate dehydrogenase complex (PDHC) were also shown to be suitable hosts for the production of pyruvate and pyruvate-derived products, l-valine, l-lysine, 2-ketoisovalerate, succinate and isobutanol (reviewed in [30]). Typically, PDHC-deficient strains are unable to grow on glucose as sole carbon source unless the medium is supplemented with acetate or ethanol [31, 32]. However, in the absence of the latter substrates, *C. glutamicum* Δ*aceE* remains metabolically active and converts glucose to pyruvate, l-alanine and l-valine under aerobic conditions [30]. Combined activities of pyruvate:quinone oxidoreductase, acetate kinase and phosphotransacetylase (Fig. 1) do not provide sufficient acetyl-CoA to replace the PDHC reaction in glucose containing medium, however, deletion of the respective *pqo* gene was shown to be beneficial for the production of pyruvate and its derived products l-valine and 2-ketoisovalerate [33, 34]. In this framework, we constructed and tested three strains as potential hosts for efficient 2,3-BD production. In addition to *C. glutamicum* Δ*ldhA*, and *C. glutamicum* Δ*aceEΔpqo*Δ*ldhA*, designed to minimize lactate and acetate formation, a third strain, *C. glutamicum* Δ*aceE*Δ*pqo*Δ*ldhA*Δ*mdh*, was constructed with an extra deletion of the malate dehydrogenase gene to reduce succinate formation (Fig. 1). In these strains we expressed *als*, *aldB*, *butA* from *L. lactis* and devised a two-stage process for efficient production of 2,3-BD. In the first stage cells were grown aerobically on acetate or glucose for biomass formation; in the second stage (production stage), biomass was used to convert glucose into 2,3-BD under non-growing and oxygen-limiting conditions.

## Results

### Design of a synthetic pathway for 2,3-BD and functional analysis

The first step in engineering *C. glutamicum* for the synthesis of 2,3-BD was to construct an artificial operon comprising *als*, *aldB*, and *butA* that form the 2,3-BD biosynthetic pathway of *L. lactis*. The three genes were cloned into pEKEx2 expression vector under control of the isopropyl β-D-1-thiogalactopyranoside (IPTG)-inducible P_tac_ promoter (Additional file 1: Fig. S1), and transformed into the three selected background strains *C. glutamicum* Δ*ldhA*, Δ*aceE*Δ*pqo*Δ*ldhA* and Δ*aceE*Δ*pqo*Δ*ldhA*Δ*mdh* (Table 1). To evaluate whether the target enzymes were functionally produced, specific activities were determined in cell extracts (Table 2). Induced expression of *als* and *aldB* genes in *C. glutamicum* Δ*ldhA*(pEKEx2-*als,aldB,butA*) led to 61- and 15-fold increase in ALS/ALDC activity when compared to control *C. glutamicum* Δ*ldh*(pEKEx2) grown on glucose and acetate, respectively; this corresponded to an increase from 0.024 ± 0.003 to 1.47 ± 0.42 U (mg of protein)^−1^ in glucose grown cells, and from 0.037 ± 0.002 to 0.54 ± 0.09 U (mg of protein)^−1^ in acetate grown cells. For *C. glutamicum* Δ*aceE*Δ*pqo*Δ*ldhA*(pEKEx2-*als,aldB,butA*), ALS/ALDC activity was increased 17-fold (0.034 ± 0.001 compared to 0.58 ± 0.12 U (mg of protein)^−1^), and for Δ*aceE*Δ*pqo*Δ*ldhA*Δ*mdh*(pEKEx2-*als,aldB,butA*) the activity was 8-fold higher (0.055 ± 0.019 compared to 0.43 ± 0.04 U (mg of protein)^−1^). The activity of BDH in strain Δ*ldhA*(pEKEx2-*als,aldB,butA*) doubled in glucose grown cells, and, unexpectedly, decreased twofold (from 0.2 to 0.1 U (mg of protein)^−1^) in acetate-grown cells, when compared to the control strain cultivated under the same conditions. Acetate grown *C. glutamicum* Δ*aceE*Δ*pqo*Δ*ldhA*(pEKEx2-*als,aldB,butA*) and Δ*aceE*Δ*pqo*Δ*ldhA*Δ*mdh*(pEKEx2-*als,aldB,butA*) showed 3- and 8-fold increase in BDH activity, compared to the control strain. Maximal specific activity values of 0.97 ± 0.19 U (mg of protein)^−1^ were obtained. In an attempt to increase BDH activity further we cloned the constitutive P_tuf_ promoter [28, 35] in front of *butA*, yielding pEKEx2-*alsaldB*P_tuf_*butA* (Additional file 1: Fig. S1). However, this increased BDH activity only slightly (Table 2).

**Table 1:** Bacterial strains and plasmids used in this study

Strains	Description	Reference
*E. coli* DH5α	Plasmid-free *E. coli*	Amersham biosciences
*C. glutamicum* ATCC13032	Wild-type strain	[60]
*C. glutamicum* Δ*ldhA*	ATCC13032 with deletion of lactate dehydrogenase gene *ldhA*	This study
*C. glutamicum* Δ*aceE*Δ*pqo*Δ*ldhA*	ATCC13032 with deletions of the genes for the E1 subunit of pyruvate dehydrogenase complex, the pyruvate:quinone oxidoreductase and the lactate dehydrogenase	[52]
*C. glutamicum* Δ*aceE*Δ*pqo* Δ*ldhA*Δ*mdh*	ATCC13032 with deletions of the genes for the E1 subunit of pyruvate dehydrogenase complex, the pyruvate:quinone oxidoreductase, lactate dehydrogenase and malate dehydrogenase	This study
*C. glutamicum* Δ*aceE*Δ*pqo* Δ*ldhA* (pEKEx2)	*C. glutamicum* Δ*aceE*Δ*pqo*Δ*ldhA* harboring plasmid pEKEx2	This study
*C. glutamicum* Δ*ldhA* (pEKEx2)	*C. glutamicum* Δ*ldhA* harboring plasmid pEKEx2	This study
*C. glutamicum* Δ*aceE*Δ*pqo* Δ*ldhA*Δ*mdh* (pEKEx2)	*C. glutamicum* Δ*aceE*Δ*pqo*Δ*ldhA*Δ*mdh* harboring plasmid pEKEx2	This study
*C. glutamicum* Δ*ldhA* (pEKEx2- *als,aldB,butA*)	*C. glutamicum* Δ*ldhA* harboring plasmid pEKEx2 *als,aldB,butA*	This study
*C. glutamicum* Δ*aceE*Δ*pqo* Δ*ldhA* (pEKEx2-*als,aldB,butA*)	*C. glutamicum* Δ*aceE*Δ*pqo*Δ*ldhA* harboring plasmid pEKEx2-*als,ald,BbutA*	This study
*C. glutamicum* Δ*aceE*Δ*pqo* Δ*ldhA*Δ*mdh* (pEKEx2-*als,aldB,butA*)	*C. glutamicum* Δ*aceE*Δ*pqo*Δ*ldhA*Δ*mdh* harboring plasmid pEKEx2-*als,aldB,butA*	This study
*C. glutamicum* Δ*aceE*Δ*pqo* Δ*ldhA* (pEKEx2-*als,aldB,*_Ptuf_ *butA*)	*C. glutamicum* Δ*aceE*Δ*pqo*Δ*ldhA* harboring plasmid pEKEx2-*als,aldB,*_Ptuf_ *butA*	This study
*C. glutamicum* Δ*aceE*Δ*pqo* Δ*ldhA*Δ*mdh* (pEKEx2-*als,aldB,*_Ptuf_ *butA*)	*C. glutamicum* Δ*aceE*Δ*pqo*Δ*ldhA*Δ*mdh* harboring plasmid pEKEx2-*als,aldB,*_Ptuf_ *butA*	This study
Plasmids		
pEKEx2	kan^R^ from pUC4 K; *P*_*trc*_ *, lacI*, pUC18 mcs, induced by addition of IPTG	[61]
pEKEx2-*als*	pEKEx2 with cloned *als* from *L. lactis*	This study
pEKEx2-*als,aldB,butA*	pEKEx2-*als* with cloned *aldB* and *butA* from *L. lactis*	This study
pEKEx2-*als,aldB,*_Ptuf_ *butA*	pEKEx2-*als* with cloned *aldB* and P_tuf_-*butA* from *L. lactis*	This study

*als* α-acetolactate synthase gene, *aldB* α-acetolactate decarboxylase gene, *butA* butanediol dehydrogenase gene, *P*_*tuf*_ a 185 bp region upstream of *tuf* gene of *C. glutamicum, kan* ^*R*^ kanamycin resistance, *P*_*trc*_ trc promoter, *lacI* lac repressor gene

**Table 2:** Overexpression and specific activities of enzymes for the synthesis of 2,3-butanediol as determined in crude cell extracts

	ALS/ALDC activity U (mg of protein)^−1^	BDH activity U (mg of protein)^−1^	ALS/ALDC over-expression (-fold)	BDH over-expression (-fold)
Δ*ldhA* (pEKEx2)*	0.02 ± 0.00	0.66 ± 0.03	–	–
Δ*ldhA* (pEKEx2-*als,aldB,butA*)*	1.47 ± 0.42	1.18 ± 0.26	61	2
Δ*ldhA* (pEKEx2)	0.04 ± 0.00	0.20 ± 0.03	–	–
Δ*ldhA* (pEKEx2-*als,aldB,butA*)	0.54 ± 0.09	0.10 ± 0.03	15	–
Δ*aceE*Δ*pqo*Δ*ldhA* (pEKEx2)	0.03 ± 0.01	0.24 ± 0.04	–	–
Δ*aceE*Δ*pqo*Δ*ldhA* (pEKEx2-*als,aldB,butA*)	0.58 ± 0.12	0.67 ± 0.01	17	3
Δ*aceE*Δ*pqo*Δ*ldhA* (pEKEx2-*als,aldB,*_Ptuf_ *butA*)	1.42 ± 0.24	0.53 ± 0.17	31	2
Δ*aceE*Δ*pqo*Δ*ldhA*Δ*mdh* (pEKEx2)	0.06 ± 0.02	0.11 ± 0.04	–	–
Δ*aceE*Δ*pqo*Δ*ldhA*Δ*mdh* (pEKEx2-*als,aldB,butA*)	0.43 ± 0.04	0.97 ± 0.19	8	8
Δ*aceE*Δ*pqo*Δ*ldhA*Δ*mdh* (pEKEx2-*als,aldB,*_Ptuf_ *butA*)	0.47 ± 0.14	1.21 ± 0.01	9	10

Cells were grown for 14 h in 2× TY medium supplemented with acetate, unless stated otherwise

Values shown are averages ± SD of at least three independent experiments and two technical replicates

*ALS* α-acetolactate synthase, *ALDC* α-acetolactate decarboxylase, *BDH* butanediol dehydrogenase

* Cells grown on glucose

### Growth profiles of engineered strains

First, we characterized growth and biomass formation of the engineered strains in shake-flasks in 2× TY medium containing glucose or acetate (Additional file 1: Fig. S2, Table S1). The Δ*aceE* strains (PDHC-deficient) are unable to grow on glucose as sole carbon source, therefore, they were grown aerobically on acetate alone. Strains *C. glutamicum* Δ*ldh*(pEKEx2) and Δ*ldhA*(pEKEx2-*als,aldB,butA*) were grown on glucose and also on acetate for comparison purposes. While the control strain *C. glutamicum* Δ*ldh*(pEKEx2) had similar specific growth rates when grown on glucose or acetate (0.52 ± 0.02 and 0.51 ± 0.05 h^−1^, respectively), Δ*ldhA*(pEKEx2-*als,aldB,butA*) displayed a lower growth rate on acetate (0.46 ± 0.01 versus 0.58 ± 0.01 h^−1^). The pH profiles were also dependent on the substrate used for growth as glucose-grown cells presented a sharper decrease in pH at the onset of stationary phase (Additional file 1: Fig. S2). The biomass formation was fairly similar for *C. glutamicum* Δ*ldh*(pEKEx2) and producer strain Δ*ldhA*(pEKEx2-*als,aldB,butA*) regardless of the substrate used for growth (maximal OD_600_ values in the range 21–24). The *C. glutamicum* Δ*aceE*Δ*pqo*Δ*ldhA*-derived producers showed growth and pH profiles identical to those of the control strain Δ*aceE*Δ*pqo*Δ*ldhA*(pEKEx2). The specific growth rate of *C. glutamicum* Δ*aceE*Δ*pqo*Δ*ldhA*Δ*mdh*(pEKEx2-*als,aldB,butA*) was higher as compared to the control strain Δ*aceE*Δ*pqo*Δ*ldhA*Δ*mdh*(pEKEx2) (0.55 ± 0.04 vs 0.46 ± 0.01 h^−1^); on the other hand, the growth rate of Δ*aceE*Δ*pqo*Δ*ldhA*Δ*mdh*(pEKEx2-*als,aldB,*_Ptuf_*butA*) was comparable to that of the control strain (0.44 ± 0.01 vs 0.46 ± 0.01 h^−1^). For all Δ*aceE*Δ*pqo*Δ*ldhA*- and Δ*aceE*Δ*pqo*Δ*ldhA*Δ*mdh*-derived strains, the initial and final pH values were 7.1 and 9.2, respectively, and maximal OD_600_ values were in the range 15–19. In summary, cell growth was not impaired by the expression of heterologous genes (Additional file 1: Table S1), reflecting the robustness of these platform strains.

### Characterization of 2,3-BD production under low-oxygen conditions

**Fig. 2:**
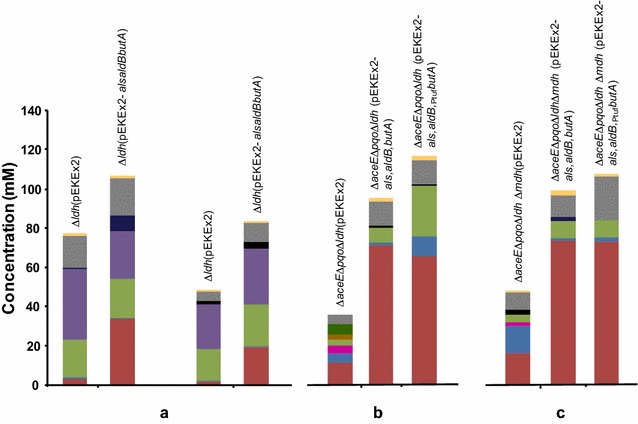
End-products of glucose metabolism in 2,3-BD producers and control strains under oxygen limiting conditions: 25 mL of cell suspension in stoppered 50-mL flasks incubated with 2 % (wt/vol) glucose for 48 h at 180 rpm and 30 °C. Control strains produced optically active 2,3-BD (most likely (*2S*,*3S*)-2,3-BD), while the engineered strains produced *meso*-2,3-BD. For simplicity, a single color is used to represent any form of 2,3-BD. Lactate dehydrogenase negative strains (**a**) grown on glucose (*left*) and acetate (*right*); triple deletion mutants *C. glutamicum* Δ*aceE*Δ*pqo*Δ*ldhA*(pEKEx2-*als,aldB,butA*), *C. glutamicum* Δ*aceE*Δ*pqo*Δ*ldhA*(pEKEx2-*als,aldB,*_Ptuf_ *butA*) (**b**) and quadruple deletion mutants *C. glutamicum* Δ*aceE*Δ*pqo*Δ*ldhA*Δ*mdh*(pEKEx2-*als,aldB,butA*) and *C. glutamicum* Δ*aceE*Δ*pqo*Δ*ldhA*Δ*mdh*(pEKEx2-*als,aldB,*_Ptuf_ *butA*) (**c**). *red* 2,3-BD; *blue* acetoin; *magenta* acetolactate; *light green* succinate; *purple* acetate; *brown* pyruvate; *dark green* α-ketoisovalerate; *black* DHA; *dark* *grey* glycerol; *yellow* l-alanine

The production of reduced compounds, such as 2,3-BD, is expected to be maximal under oxygen limiting conditions. Having this in mind, we devised a strategy for the production of 2,3-BD in which aerobically grown cells are collected, washed, re-suspended in minimal medium and provided with glucose under oxygen limiting conditions. Oxygen concentration was not measured, but the observed formation of organic acids indicated restricted oxygen availability (Fig. 2).

In a first attempt to reduce pyruvate consuming reactions leading to end-products other than 2,3-BD, we used as background *C. glutamicum* Δ*ldhA*. During the production phase, glucose-grown cells produced 2,3-BD with a yield of 0.05 mol per mol of glucose (Table 3, Additional file 1: Table S3); the yield was even lower for cells grown on acetate (0.02 mol per mol of glucose). The glucose consumption rate (GCR) was low, i.e., 2.5 ± 0.4 nmol min^−1^ mg CDW^−1^. For comparison purposes, the GCR of the wild type strain (grown on acetate) was determined in a similar experimental set-up to give 24.6 ± 0.5 nmol min^−1^ mg CDW^−1^. Actually, this value agrees perfectly with 25 nmol min^−1^ mg CDW^−1^, the GCR reported earlier for wild type *C. glutamicum* with a different experimental set-up [36]. Importantly, the introduction of the lactoccocal pathway had a positive effect on GCR in glucose grown *C. glutamicum* Δ*ldhA*(pEKEx2-*als,aldB,butA*) (4.4 ± 0.4 nmol min^−1^ mg CDW^−1^ compared to 2.5 ± 0.4 nmol min^−1^ mg CDW^−1^ in the control strain), and no effect on acetate grown cells (3.4 ± 0.4 compared to 3.0 ± 0.2 nmol min^−1^ mg CDW^−1^ in the control strain). 2,3-BD produced by the engineered strain Δ*ldhA*(pEKEx2-*als,aldB,butA*) grown on glucose and acetate was, respectively, 34 ± 3 mM (yield of 0.34 ± 0.04 mol per mol glucose, a sevenfold increase compared to the control strain), and 19.5 ± 0.7 mM (0.27 ± 0.06 mol per mol glucose; 11-fold increase). Other major products of metabolism were acetate, succinate, and glycerol at respective concentrations of 29 ± 2, 22 ± 2, 10 ± 1 mM in cells grown on acetate, and 25 ± 6, 20 ± 1, 19 ± 6 mM in glucose grown cells (Fig. 2, Additional file 1: Table S3).

**Table 3:** Glucose (Glc) consumption rate (GCR), molar yield and productivity of 2,3-butanediol (2,3-BD), and carbon recovery (CR) by the control and engineered *C. glutamicum* strains

	GCR (nmol min^−1^ mg CDW^−1^)	Yield (mol 2,3-BD per mol Glc)	Productivity (nmol min^−1^ mg CDW^−1^)	CR (%)
Δ*ldhA* (pEKEx2)*	2.5 ± 0.4	0.05 ± 0.00^#^	0.12 ± 0.01	81 ± 3
Δ*ldhA* (pEKEx2- *als,aldB,butA*)*	4.4 ± 0.4	0.34 ± 0.04	1.28 ± 0.09	79 ± 9
Δ*ldhA* (pEKEx2)	3.0 ± 0.2	0.02 ± 0.01^#^	0.06 ± 0.03	77 ± 1
Δ*ldhA* (pEKEx2- *als,aldB,butA*)	3.4 ± 0.4	0.27 ± 0.06	0.53 ± 0.13	82 ± 3
Δ*aceE*Δ*pqo*Δ*ldhA* (pEKEx2)	1.6 ± 0.3	0.22 ± 0.01^#^	0.30 ± 0.00	87 ± 1
Δ*aceE*Δ*pqo*Δ*ldhA* (pEKEx2-*als,aldB,butA*)	5.4 ± 0.7	0.66 ± 0.05	3.55 ± 0.58	84 ± 7
Δ*aceE*Δ*pqo*Δ*ldhA* (pEKEx2-*als,aldB,*_Ptuf_ *butA*)	6.1 ± 0.1	0.61 ± 0.06	3.13 ± 0.22	87 ± 3
Δ*aceE*Δ*pqo*Δ*ldhA*Δ*mdh* (pEKEx2)	1.6 ± 0.3	0.34 ± 0.05^#^	0.53 ± 0.00	91 ± 6
Δ*aceE*Δ*pqo*Δ*ldhA*Δ*mdh* (pEKEx2-*als,aldB,butA*)	6.0 ± 0.3	0.64 ± 0.09	4.34 ± 0.16	90 ± 4
Δ*aceE*Δ*pqo*Δ*ldhA*Δ*mdh* (pEKEx2-*als,aldB,*_Ptuf_ *butA*)	6.5 ± 0.5	0.64 ± 0.10	4.25 ± 0.41	91 ± 7

In the first phase, cells were grown aerobically on 1 % (wt/vol) potassium acetate except for the strains indicated with an asterisk for which 0.5 % (wt/vol) glucose was used instead. Second phase fermentations were carried out for 48 h, using 25 mL of cell suspension in closed 50-mL falcon flasks with glucose as substrate. The cardinal symbol indicates that optically active 2,3-BD was produced; remaining strains produced *meso*-form of 2,3-BD. Values are averages of at least three independent experiments. A single NMR spectrum was acquired for each sample

The inactivation of the PDHC and PQO in the producer strain Δ*aceE*Δ*pqo*Δ*ldhA*(pEKEx2-*als,aldB,butA*) had a drastic effect on 2,3-BD yield that increased from 0.27 ± 0.06 in Δ*ldhA*(pEKEx2-*als,aldB,butA*), to 0.66 ± 0.05 mol per mol, reflecting primarily the reduction of acetate formation (from 29 mM to below 0.5 mM). Moreover, the GCR increased about 1.6-fold and there was a sevenfold increase in overall productivity. Glycerol and succinate were the major side-products (12 ± 1 and 8 ± 1 mM, respectively) (Fig. 2).

Further engineering aimed at succinate reduction (MDH inactivation by deletion of the cg2613-encoded activity), left both yield and GCR essentially unchanged while productivity was slightly improved in *C. glutamicum* Δ*aceE*Δ*pqo*Δ*ldhA*Δ*mdh*(pEKEx2-*als,aldB,butA*) (4.3 ± 0.2 compared to 3.6 ± 0.6 nmol min^−1^ mg CDW^−1^). Strains in which the lactococcal *butA* gene was placed under the control of P_tuf_ promoter showed similar behavior to those which had this gene under P_tac_ control in terms of all production parameters (Table 3; Fig. 2). In summary, *C. glutamicum* Δ*aceE*Δ*pqo*Δ*ldhA*(pEKEx2-*als,aldB,butA*), Δ*aceE*Δ*pqo*Δ*ldhA*Δ*mdh*(pEKEx2-*als,aldB,butA*) and Δ*aceE*Δ*pqo*Δ*ldhA*Δ*mdh*(pEKEx2-*als,aldB,*_Ptuf_*butA*) showed the highest yield of about 0.65 mol 2,3-BD per mol of glucose with the latter two strains exhibiting the highest productivities of about 4.3 nmol 2,3-BD per min and mg CDW (Table 3). All control strains produced optically active 2,3-BD, while only the *meso*-form was found as end-product of the producer strains (Fig. 2, Additional file 1: Table S3).

It is important to note that pH was not controlled during the production phase. All fermentations started at pH 7.0, but the final pH value varied as mutants exhibited different acidifying properties due to their differential ability to produce organic acids. At the end of 48 h-fermentation, pH was between 5.1 and 5.3 for *ldhA*-negative strains. As expected, reduction of succinate and acetate, and accumulation of neutral compounds led to higher final pH values: 6.6, 6.4, 5.7 and 6.6 for *C. glutamicum* Δ*aceE*Δ*pqo*Δ*ldhA*(pEKEx2-*als,aldB,butA*), Δ*aceE*Δ*pqo*Δ*ldhA*Δ*mdh*(pEKEx2-*als,aldB,butA*), Δ*aceE*Δ*pqo*Δ*ldhA*(pEKEx2-*als,aldB,*_Ptuf_*butA*) and Δ*aceE*Δ*pqo*Δ*ldhA*Δ*mdh*(pEKEx2-*als,aldB,*_Ptuf_*butA*), respectively. Earlier work has shown that a decrease in pH results in considerably lower GCR in resting cells of wild-type *C. glutamicum* [36].

### Optimization of 2,3-BD production by manipulation of oxygen supply

**Fig. 3:**
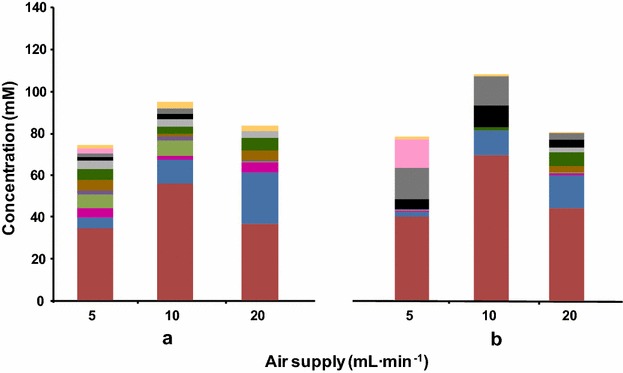
Effect of aeration rate on end-products of glucose metabolism in the triple deletion mutant *C. glutamicum* Δ*aceE*Δ*pqo*Δ*ldhA*(pEKEx2-*als,aldB,*_Ptuf_ *butA*) (**a**) and the quadruple deletion mutant Δ*aceE*Δ*pqo*Δ*ldhA*Δ*mdhA* (pEKEx2-*als,aldB,*_Ptuf_ *butA*) (**b**). Fifty mL of cell suspension in 80-mL fermenter incubated with 2 % (wt/vol) glucose for 30 h at 30 °C. The air flow was 5, 10 or 20 mL/min. *Red* 2,3-BD; *blue* acetoin; *magenta* acetolactate; *light green* succinate; *purple* acetate; *brown* pyruvate; *dark green* α-ketoisovalerate; *light grey* α-ketoglutarate; *black* DHA; *dark grey* glycerol; *pink* ethanol; *yellow* l-alanine

Engineered strains of *C. glutamicum* converted up to two-thirds of glucose into 2,3-BD (Table 3), however the consumption rate of glucose was not satisfactory when compared to other *C. glutamicum* strains, such as those manipulated for the production of isobutanol and succinate [20, 28]. The accumulation of reduced compounds, such as succinate and glycerol, hinted that the low GCR in the engineered strains could result from a high NADH:NAD^+^ ratio, as previously proposed [37]. Oxygen can be an alternative acceptor of electrons from NADH, via the electron transfer chain, hence we performed fermentations under different controlled oxygen supply conditions (sparging rates of 5, 10 or 20 mL air min^−1^) in the mini-fermenter as described in Methods, to determine the conditions that would support maximal yield, GCR and productivity (Table 4, Additional file 1: Table S4, Fig. 3). Strains *C. glutamicum* Δ*aceE*Δ*pqo*Δ*ldhA*(pEKEx2-*als,aldB,*_Ptuf_*butA*) and Δ*aceE*Δ*pqo*Δ*ldhA*Δ*mdh*(pEKEx2-*als,aldB,*_Ptuf_*butA*) were selected for this optimization step as they showed particularly high ALS/ALDC and BDH activity, respectively.

**Table 4:** Glucose (Glc) consumption rate (GCR), molar yield and productivity of 2,3-butanediol (2,3-BD), and carbon recovery (CR) achieved with *C. glutamicum* Δ*aceE*Δ*pqo*Δ*ldhA*(pEKEx2-*als,aldB,*_Ptuf_ *butA*) and *C. glutamicum* Δ*aceE*Δ*pqo*Δ*ldhA*Δ*mdh*(pEKEx2-*als,aldB,*_Ptuf_ *butA*) strains under different aeration conditions

	Airflow (mL min^−1^)	GCR (nmol min^−1^ mg CDW^−1^)	Yield (mol 2,3-BD per mol Glc)	Productivity (nmol min^−1^ mg CDW^−1^)	CR (%)
Δ*aceE*Δ*pqo*Δ*ldhA* (pEKEx2-a*ls,aldB,*_Ptuf_ *butA*)	5	7.2 ± 0.6	0.44 ± 0.04	3.4 ± 0.3	89 ± 6
	10	15.8 ± 1.7	0.57 ± 0.03	8.1 ± 0.4	91 ± 4
	20	14.7 ± 1.1	0.36 ± 0.06	5.0 ± 0.9	83 ± 10
Δ*aceE*Δ*pqo*Δ*ldhA*Δ*mdh* (pEKEx2-a*ls,aldB,*_Ptuf_ *butA*)	5	11.1 ± 1.0	0.52 ± 0.03	5.5 ± 0.7	85 ± 2
	10	21.1 ± 0.8	0.66 ± 0.01	10.9 ± 1.8	90 ± 8
	20	22.8 ± 1.6	0.46 ± 0.02	8.0 ± 0.7	78 ± 7

Cells were grown aerobically on 1 % (wt/vol) potassium acetate. The second, production phase was performed in a 80-mL fermenter at 30 °C for 30 h with glucose as substrate. The cell suspension was sparged with air at the flow rates indicated. *Meso*-2,3-BD was by far the major stereoisomer. At the optimal aeration rate (10 mL min^−1^), there was 95 % of the *meso*-form. Values are averages of three independent experiments; a single NMR spectrum was acquired for each sample

**Fig. 4:**
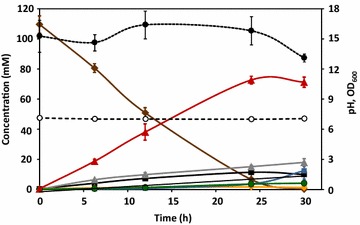
Fermentation profile of the best 2,3-BD producer strain *C. glutamicum* Δ*aceE*Δ*pqo*Δ*ldhA*Δ*mdh*(pEKEx2-*als,aldB,*_Ptuf_ *butA*) as a function of time. Fifty mL of cell suspension in 80-mL fermenter incubated with 2 % (wt/vol) glucose for 30 h at 30 °C. The air flow was 10 mL min^−1^. *Error bars* are shown as standard deviation of three independent replicates. *Black coloured circle*, biomass as OD_600_; *white coloured circle* pH value; red coloured triangle, 2,3-BD; *blue coloured square*, acetoin; *brown coloured diamond* glucose; *green coloured circle* α-ketoisovalerate; *black coloured square* DHA; *grey coloured triangle* glycerol; *yellow coloured diamond* l-alanine

**Fig. 5:**
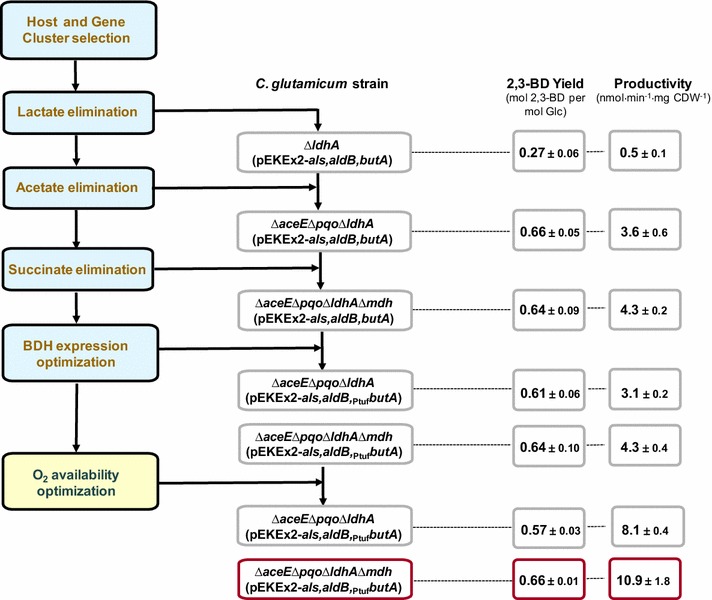
A summary of the stepwise systematic approach used to engineering *C. glutamicum* for the production of 2,3-BD. The values reflect the impact of the several steps on yield and productivity. *Blue boxes* refer to strain optimization steps, while the *yellow box* indicates the process optimization step. *2,3-BD* 2,3-butanediol, *Glc* glucose

Cell suspensions of *C. glutamicum* Δ*aceE*Δ*pqo*Δ*ldhA*(pEKEx2-*als,aldB,*_Ptuf_*butA*) and Δ*aceE*Δ*pqo*Δ*ldhA*Δ*mdh*(pEKEx2-*als,aldB,*_Ptuf_*butA*) under constant air flow of 5 mL min^−1^ showed an increased GCR [7.2 ± 0.6 and 11.1 ± 1.0 nmol min^−1^ mg CDW^−1^ in mini-fermenter for the two respective strains (Table 4), compared to 6.1 ± 0.1 and 6.5 ± 0.5 nmol min^−1^ mg CDW^−1^ in flasks (Table 3)]; the productivity in mini-fermenter was 3.4 ± 0.3 and 5.5 ± 0.7 nmol min^−1^ mg CDW^−1^ compared to 3.1 ± 0.2 and 4.3 ± 0.4 nmol min^−1^ mg CDW^−1^ in flasks, but the yield was higher for fermentations in closed flasks (Tables 3, 4). Upon doubling of the air flow to 10 mL min^−1^, GCR and the productivity of *C. glutamicum* Δ*aceE*Δ*pqo*Δ*ldhA*(pEKEx2-*als,aldB,*_Ptuf_*butA*) and Δ*aceE*Δ*pqo*Δ*ldhA*Δ*mdh*(pEKEx2-*als,aldB,*_Ptuf_*butA*) increased two-fold; both strains produced 2,3-BD at highest yields (0.57 ± 0.03 and 0.66 ± 0.01 mol 2,3-BD per mol glucose). Interestingly, further increase in the flow rate to 20 mL min^−1^ resulted in significantly lower yields and productivities (Table 4). Ethanol was produced by both strains in experiments using 5 mL min^−1^ air, and glycerol was absent only in the experiment using 20 mL min^−1^ air with the Δ*aceE*Δ*pqo*Δ*ldhA*(pEKEx2-*als,aldB,*_Ptuf_*butA*) strain. Other side products in these experiments were acetoin, succinate, dihydroxyacetone (DHA), pyruvate, acetate, l-alanine, α-acetolactate, α-ketoglutarate, and α-ketoisovalerate (Fig. 3, Additional file 1: Table S4). Among these, acetoin formation showed a clear dependence on oxygen availability, increasing about 3- and 5-fold when the air flow was increased from 5 to 10 mL min^−1^ and from 5 to 20 mL min^−1^, respectively. Dissolved oxygen was not controlled, but we confirmed that the oxygen concentration was below the detection limit of the oxygen electrode even at the highest aeration rate. The time course for glucose consumption and end-product formation is illustrated in Fig. 4 for the best producer strain. In summary, under an air flow of 10 mL min^−1^*C. glutamicum* Δ*aceE*Δ*pqo*Δ*ldhA*Δ*mdh*(pEKEx2-*als,aldB,*_Ptuf_*butA*) produced 70 ± 8 mM 2,3-BD with a yield of 0.66 mol per mol of glucose and productivity of 11 nmol min^−1^ mg CDW^−1^, which represent a notable improvement (Fig. 5).

## Discussion

The wild-type *C. glutamicum* produces vestigial amounts of 2,3-BD [38]. Accordingly, low levels of 2,3-BD were also found in the end-products of glucose metabolism by the control strain *C. glutamicum* Δ*ldhA*(pEKEx2), the initial host in this work (Table 3). *C. glutamicum* was the favored candidate for 2,3-BD production given its high performance as an industrial organism and its GRAS status. Moreover, we verified that 2,3-BD has low toxicity to *C. glutamicum* as cell growth was not affected by 2 % 2,3-BD (data not shown). 2,3-BD producing bacteria, such as *Klebsiella spp*., synthesize this diol from pyruvate in a sequence of three reactions catalyzed by α-acetolactate synthase, α-acetolactate decarboxylase and butanediol dehydrogenase. In this work, we constructed an artificial operon with the three relevant genes of *L. lactis* and introduced this heterologous pathway into *C. glutamicum* (Fig. 1).

*C. glutamicum* is able to synthesize α-acetolactate from pyruvate via the action of the anabolic enzyme, AHAS; however, this enzyme is highly inhibited by the branched chain amino acids derived from α-acetolactate [39]. Therefore, our engineering strategy endowed the host strain with a heterologous ALS, i.e., the catabolic enzyme from *L. lactis*, which is not susceptible to amino acid inhibition [40]. The enhanced activity of ALS should also be beneficial for directing the flux towards 2,3-BD.

The second step in the biosynthetic pathway, decarboxylation of α-acetolactate into *R*-acetoin, is carried out by α-acetolactate decarboxylase. A homologue of this enzyme is not predicted in the genome of *C. glutamicum*, and indeed the ALS/ALDC activity was low in cell extracts of all control strains (Table 2). The third step, reduction of acetoin, is performed by BDH, and a relatively high activity (0.2–0.3 U(mg protein)^−1^) was detected in the control strains (Table 2). Actually, this is not surprising given that *C. glutamicum* possesses (*2S,3S*)-BDH, which is claimed to be absolutely stereospecific for *S*-acetoin [5]. Like all known ALDCs, the enzyme from *L. lactis* produces exclusively *R*-acetoin [41], thus a BDH with suitable stereospecific properties needed to be included.

^1^H-NMR can distinguish the optically active from the *meso*-form of 2,3-BD (Additional file 1: Fig. S3). Control strains produced only optically active 2,3-BD (Table 3), most likely derived from diacetyl formed via spontaneous decarboxylation of α-acetolactate in the presence of oxygen. Diacetyl is then converted to 2,3-(*2S,3S*)-BD in two reduction reactions catalyzed by the endogenous BDH (Fig. 1). In contrast to the control strains, *meso*-2,3-BD was by far the main form synthesized by the producer strains, the optically active form (around 5 % of total 2,3-BD), being detected only in fermentations performed at the highest aeration rates, in agreement with the oxygen dependence of the spontaneous decarboxylation of α-acetolactate. Given that *C. glutamicum* lacks α-acetolactate decarboxylase, the presence of BDH in this bacterium is rather puzzling. Moreover, according to a recent study, *C. glutamicum* BDH is a promiscuous enzyme that besides acetoin recognizes DHA as substrate, though with a low affinity (30 mM or higher DHA concentration needed for activity) [42]. Further work should be directed to clarify the physiological role of BDH in *C. glutamicum.*

The *L. lactis* genes (*als*, *aldB*, *butA*) were functionally expressed in the three host strains. The combined activity ALS/ALDC was in the range 0.4–1.4 U (mg of protein)^−1^ for producer strains grown on acetate, corresponding to overexpression levels of 8–31-fold. Opportunely, the heterologous activities are comparable to those of glycolytic enzymes measured in crude extracts of *C. glutamicum* [43]. Overexpression of *butA* (encoding BDH activity) was similarly successful, yielding final BDH activity up to 1.2 U (mg of protein)^−1^, which confirms the suitability of using the lactococcal genes for 2,3-BD production in *C. glutamicum*.

The introduction of the assembled pathway in *C. glutamicum* Δ*ldhA* enabled efficient redirection of the flux from pyruvate into 2,3-BD. However, the yield was relatively low (0.3 mol per mol glucose) and the two major products were succinate and acetate. Hence, the next engineering step had the objective of suppressing acetate pathways; this proved to be crucial in the by-product elimination strategy, as the 2,3-BD yield doubled in the Δ*aceE*Δ*pqo*Δ*ldhA*-derived producer strains (Table 3; Fig. 5). Additional inactivation of *mdh* did not improve the yield significantly, but there was a slight increase in productivity (Table 3).

Curiously, the strains derived from *C. glutamicum* Δ*aceE*Δ*pqo*Δ*ldhA*Δ*mdh* produced substantial amounts of succinate despite the intended suppression of succinate formation via the reductive branch of the TCA cycle [36]. The genome of *C. glutamicum* includes *mdhB* (cg0763), putatively assigned as malate/L-lactate dehydrogenase, hence this second activity may contribute to succinate formation. On the other hand, involvement of the glyoxylate shunt and/or the oxidative branch of the TCA cycle cannot be ruled out.

Glycerol was the second major side-product detected in *C. glutamicum* Δ*aceE*Δ*pqo*Δ*ldhA*- and Δ*aceE*Δ*pqo*Δ*ldhA*Δ*mdh*-derived strains. Two pathways have been proposed for the synthesis of this polyol in *C. glutamicum* [42, 44]. Jojima and coworkers [42] proposed that under oxygen-deprivation conditions glycerol is formed through reduction of DHA by the activity of endogenous BDH. On the other hand, DHA is a product of dihydroxyacetone-phosphate (DHAP) dephosphorylation via the respective phosphatase [45]. Therefore, endogenous BDH and DHAP phosphatase are obvious targets for inactivation in further attempts to minimize side products. In this way, the electrons that are used for glycerol formation could be directed to acetoin reduction. The elimination of BDH would also be advantageous for the stereochemical purity of the desired end-product, since the small contamination with the optically active form would vanish, though at the expense of some carbon loss in the form of diacetyl.

Under production conditions (oxygen limitation), it is anticipated that the BDH activity plays an important role in satisfying the redox balance via cofactor recycling. Further enhancement of BDH activity was attempted by including an additional promoter, but the BDH activity remained unchanged (Tables 2, 3). However, a high BDH activity would not fix the redox imbalance since the synthesis of 2,3-BD results in net production of 1 mol NADH per mol of glucose consumed, and an additional sink for the reducing power becomes mandatory. Accordingly, providing controlled amounts of oxygen significantly improved GCR and 2,3-BD productivity.

Manipulation of oxygen availability had no clear impact on 2,3-BD yield, but was crucial to increase GCR, which reached maximal values of approx. 21 nmol min^−1^ mg CDW^−1^, comparable to those of *C. glutamicum* strains engineered for isobutanol production [20]. This optimization step was more effective for *C. glutamicum* Δ*aceE*Δ*pqo*Δ*ldhA*Δ*mdh*(pEKEx2-*als,aldB,*_Ptuf_*butA*) than for Δ*aceE*Δ*pqo*Δ*ldhA*(pEKEx2-*als,aldB,*_Ptuf_*butA*) (Table 4). The reason could be related with the different BDH activity in these two strains (Table 2). The stronger activity in *C. glutamicum* Δ*aceE*Δ*pqo*Δ*ldhA*Δ*mdh*(pEKEx2-*als,aldB,*_Ptuf_*butA*) would pull the flux towards 2,3-BD more efficiently, with concomitant benefit on the rate of NAD^+^ regeneration, and hence on the GCR. Further increase in the air supply (from 10 to 20 mL min^−1^) had a negative impact on 2,3-BD yield, which is in conformity with the equivalent accumulation of acetoin. This shift in end-products towards compounds less reduced than 2,3-BD indicates that the competition of oxygen as an electron sink became excessive.

Thus far, *Klebsiella* spp., *E. aerogenes* and *S. marcescens* are the top producers of 2,3-BD (reviewed in [46]; see also Additional file 1: Table S5 and references therein, [47–49]), however, the pathogenicity of these strains is regarded as a disadvantage and hinders large-scale production. The best non-pathogenic producer is *Paenibacillus polymyxa* (72 g L^−1^ and about 2 g L^−1^ h^−1^), which grows in complex medium and has the drawback of producing exopolysaccharides that increase the viscosity of the fermentation broth [13]. Engineered *S. cerevisiae* has a 0.29 g L^−1^ h^−1^ productivity and a yield of 0.70 mol 2,3-BD per mol glucose, while engineered *E. coli* produces 1.19 g L^−1^ h^−1^ with a yield of 0.82 mol mol^−1^ [14, 15]. The theoretical maximal yield of 2,3-BD from glucose is 1 mol per mol (0.5 g per g), and it was almost reached with pathogenic (*Klebsiella* spp.), and non-pathogenic producers (*Bacillus* spp.) (Additional file 1: Table S5 and references therein). Compared with these non-native producers, the best *C. glutamicum* 2,3-BD producer, Δ*aceE*Δ*pqo*Δ*ldhA*Δ*mdh*(pEKEx2-*als,aldB,*_Ptuf_*butA*), has a productivity close to the lower limit (10.9 nmol min^−1^ mg CDW^−1^ corresponding to 0.21 g L^−1^ h^−1^), and a good yield (0.66 mol 2,3-BD per mol glucose). Our work used cell suspensions at a relatively low OD_600_ (20–30, corresponding to about 5 g CDW L^−1^), but *C. glutamicum* can easily be grown to much higher cell density, allowing for higher 2,3-BD productivity during the production phase. For example, the utilization of a high density bioreactor (50 g CDW L^−1^), as in [29], would lead to one order of magnitude greater productivity (around 2 g L^−1^ h^−1^), which compares well with that of the non-pathogenic natural producer [13]. Further increase in GCR of engineered *C. glutamicum* strains might be possible with a more fine manipulation of the oxygen supply conditions.

While this manuscript was in a late stage of preparation, a study was published by Yang et al. [50] with the same purpose as ours, albeit using different engineering strategies, host strains and production process. The 2,3-BD yield obtained was lower than that presented here (0.47 vs 0.66 mol per mol glucose), but a more extended comparison is hampered by the very different production processes used in the two studies.

## Conclusions

By using a systematic approach *C. glutamicum* was successfully engineered for the production of 2,3-BD (Fig. 5). Organic acid production was strongly reduced or eliminated and adequate activities of the 2,3-BD biosynthetic pathway were achieved. By means of genetic engineering and manipulation of production conditions, productivity and yield were optimized. Characterization of production provided insight into metabolic features of producer strains, and indicated directions for further rounds of strain improvement. Future work should aim at increasing 2,3-BD yield by eliminating the side-products DHA and glycerol. By using the full potential of *C. glutamicum*, improvement of productivity and titer is also feasible. Additionally, we propose that the optimized construct, under adequate aeration conditions, can be used as a GRAS-platform for production of acetoin, another valuable chemical widespread in the food industry as flavor enhancer. Alternatively, straightforward developments, e.g., omission of the *butA* gene in the artificial operon and disruption of endogenous BDH, are expected to yield efficient acetoin producers.

## Methods

### Bacterial strains and growth conditions

Strains and plasmids used in this work are shown in Table 1. Cells were grown aerobically in the 2× TY medium described by Sambrook and Green [51]. Medium was supplemented either with 0.5 % (wt/vol) glucose or with 1 % (wt/vol) potassium acetate. Plasmid-harbouring strains were selected using kanamycin (25 μg mL^−1^). Gene expression was induced by using 0.5 mM isopropyl β-d-1-thiogalactopyranoside (IPTG). Cultivations were performed at 30 °C with constant agitation at 160 rpm and were initiated by addition of a pre-culture to an optical density at 600 nm (OD_600_) of about 0.3. Growth was monitored by measuring the OD_600_. Specific growth rates (μ) were calculated through linear regression of the plots of ln(OD_600_) *versus* time during the exponential growth phase. For growth characterization, samples (1 mL) were taken periodically and pH was measured.

For the molecular biology procedures, *E. coli* DH5α was grown in test tubes containing 5 mL of 2× TY medium at 37 °C and 180 rpm. Plasmid selection was achieved using 50 μg kanamycin per mL.

### Construction of strains and plasmids

All primers used are listed in Additional file 1: Table S2. To construct control strain *C. glutamicum* ∆*ldhA*, deletion of *ldhA* (cg3219) gene was performed according to [52], using the primer pairs described there. Deletion of the *mdh* (cg2613) gene in *C. glutamicum* ∆*aceE*∆*pqo*∆*ldhA* (*aceE*, cg2466; *pqo*, cg2891; [52]) was performed as described in [20]. Chromosomal DNA of *L. lactis* MG1363, isolated according to [53], was used as template in PCR amplifications. In the first cloning step, the α-acetolactate synthase gene (*als*) was amplified from *L. lactis* chromosome using primer pair als-FW and als-RE and cloned into plasmid pEKEx2. In the second step, genes encoding the α-acetolactate decarboxylase (*aldB*) and 2,3-butanediol dehydrogenase (*butA*) of *L. lactis* were amplified using primers aldB-FW, aldB-RE, butA-FW and butA-RE, ligated, and subsequently inserted into pEKEx2-*als*, giving pEKEx2-*als,aldB,butA*. Alternatively, gene *butA* fused with 185 bp region upstream of *tuf* gene (P_tuf_) was synthesized by NZYTech (Lisbon, Portugal). Plasmid pUC57 containing P_tuf_-*butA* fusion was amplified in *E. coli* DH5α and restricted using *BamH*I and *Kpn*I. The P_tuf_-*butA* fragment was purified from agarose gel and ligated to *aldB*, after which joint DNA fragment was inserted into pEKEx2-*als*, yielding pEKEx2-*als,aldB,*_Ptuf_*butA.* Plasmids were obtained and maintained in *E. coli* DH5α. After verification of the correctness of the insert by sequencing (GATC, Konstanz, Germany), plasmids were isolated from *E. coli* and electroporated into competent *C. glutamicum* cells using the procedure described in [54] and [55]. Then, plasmids were isolated from *C. glutamicum* in the following way: 5 mL of overnight culture was centrifuged for 1 min at 16,100×*g* and room temperature, to be re-suspended in 0.2 mL of solution A (50 mM glucose, 25 mM Tris–HCl, 10 mM EDTA, pH 8.0, freshly prepared). One μL of RNAse A (10 mg mL^−1^), and 15 mg mL^−1^ (final concentration) lysozyme were added, and the lysis was performed for 1.5 h at 55 °C. After the initial lysis step, isolation was continued with comercially available kits according to supplier instructions (Qiagen MiniPrep or Illustra Plasmid Mini Preparation Kit, GE Healthcare). Isolated plasmids were used as templates for PCR amplification of the inserted genes. Amplified region sequence was confirmed to be correct by sequencing (GATC, Konstanz, Germany).

### Preparation of cell suspensions

Cells grown as described above were harvested 14 h after inoculation, centrifuged (3214×*g*, 10 min, 4 °C), and washed once with 0.9 % (wt/vol) NaCl. The resulting cell suspension was centrifuged (3214×*g*, 10 min, 4 °C) and the pellet was re-suspended in a minimal CGXII basal solution at pH 7.0, that contained per liter: 1 g KH_2_PO_4_, 1 g K_2_HPO_4_, 21 g MOPS (3-[N-morpholino] propanesulfonic acid), 0.25 g MgSO_4_·7 H_2_O, 10 mg CaCl_2_·2 H_2_O [56]. Nitrogen sources (urea and (NH_4_)_2_SO_4_) were omitted, in order to minimize the biosynthesis of amino acids during the production phase.

### Two-stage fermentations processes

*C. glutamicum* strains were grown and cell suspensions prepared as described above. For the second fermentation stage (production phase), 50 mL of cell suspension (OD_600_ of 15–20) were placed in an 80-mL fermenter (mini-fermenter), kept at 30 °C with a water bath and mixed with a magnetic stirrer at 200 rpm. Aeration was performed by sparging sterile air at a rate of 5, 10 or 20 mL min^−1^. The oxygen electrode (InPro^®^ 6100/320/T/N, Mettler Toledo), was used to attempt monitoring dissolved oxygen during the production stage. The electrode was calibrated to 0 and 100 % after equilibration with pure argon and air, respectively. Glucose was provided to a final concentration of 2 % (wt/vol) and samples were collected over a period of 30 h. For the first round evaluation of the performance of the several constructs, 25 mL of concentrated cell suspension (OD_600_ of between 20 and 30) were placed in 50 mL closed falcon tubes kept at 30 °C in a rotary shaker (180 rpm). Glucose was provided to a final concentration of 2 % (wt/vol) and the fermentation was allowed to proceed for 48 h. Samples (1 mL) were centrifuged for 5 min at room temperature and 16,100×*g*; supernatants were then separated and kept at −20 °C until further analysis. End-products of metabolism were quantified by using ^1^H-NMR.

### Enzymatic activities

*C. glutamicum* strains were grown as described above and cell suspensions prepared in 50 mM PIPES at pH 7.0. Crude cell extracts were prepared by using glass beads (<106 μm) in a MiniBeadbeater-8 cell disrupter (Biospec Products) in 3 cycles of 1 min, with breaks of 3 min during which the extracts were kept on ice. After disruption, extracts were centrifuged at 16,100×*g* for 20 min at 4 °C. All enzyme activities were assayed at 30 °C in a Beckman Coulter DU 800 spectrophotometer in 50 mM PIPES (pH 7.0). One unit of enzyme activity corresponds to 1 μmol of substrate/product converted/formed per minute under the experimental conditions applied. The protein concentration was determined using Pierce BCA Protein Assay Kit (Thermo Scientific). Specific activity was expressed as units (μmol min^−1^) per milligram of protein [U (mg of protein)^−1^]. α-Acetolactate synthase activity was determined as described by [57]. The reaction was stopped by the addition of 100 μL of 6 N H_2_SO_4_, and acetoin was quantified colorimetrically at 525 nm as described by Westerfeld [58]. Butanediol dehydrogenases were assayed as described by Stormer [59], in a modified reaction mixture containing 50 mM buffer, 0.2 mM NADH and 2.5 mM racemic acetoin.

### NMR spectroscopy

All ^1^H-NMR spectra were acquired on a Bruker AVANCE II + 400 MHz spectrometer (Bruker BioSpin GmbH) at 27 °C, using a BBO-F probe head. Pre-saturation of the residual water signal was applied. Acquisition parameters: flip angle of 90°; 32 K acquisition data points; repetition delay of 31.5 s. Formate was used as a concentration standard. The high reproducibility of the NMR measurements is illustrated in Additional file 1: Fig. S4 to justify the acquisition of a single spectrum for each biological replicate (Tables 3, 4).

### Chemicals and reagents

For the molecular biology purposes, RNAse A and Pwo polymerase were purchased from Roche Life Science; lysozyme (Fluka) was purchased from Sigma-Aldrich, while all other enzymes were from New England Biolabs. Acetoin used in enzymatic assays was from Sigma-Aldrich. All other chemicals were commercially available reagent-grade (Sigma-Aldrich or Merck Sharp & Dohme).

## Additional file

10.1186/s12934-015-0362-x **Figure S1.** Plasmid maps of pEKEx2-*als,aldB,butA* and of pEKEx2-*als,aldB,*_Ptuf_ *butA*. **Figure S2.** Growth curves and pH profiles of the 2,3-butanediol producer and parental strains, grown in 2×TY medium for 30 h at 160 rpm and 30 °C. **Figure S3.** ^1^H-NMR spectra of end-products of glucose metabolism in 2,3-butanediol producers. **Figure S4.** ^1^H-NMR spectra of end-products of glucose metabolism by wild type *C. glutamicum* under oxygen limiting conditions to illustrate the high reproducibility of the NMR measurements. **Table S1.** Growth parameters of producer strains as compared to the control strains. **Table S2.** Primers used in this study. **Table S3.** End-products of glucose metabolism and residual glucose measured in supernatants of cell suspensions of parent and producer strains incubated under oxygen limiting conditions (closed falcon tubes) for 48 h. **Table S4.** End-products of glucose metabolism and residual glucose measured in supernatants of cell suspensions of producer strains incubated under different aeration conditions for 30 h. **Table S5.** Summary of the best microbial 2,3-butanediol producers.
