# Supplementary material for: Engineering Corynebacterium glutamicum for the production of 2,3-butanediol
Source: Microb Cell Fact. 2015 Oct 29;14:171. doi: 10.1186/s12934-015-0362-x (PMC4625470; doi:10.1186/s12934-015-0362-x)
Supplement: Supplementary file 1 — 10.1186/s12934-015-0362-x Figure S1. Plasmid maps of pEKEx2-als,aldB,butA and of pEKEx2-als,aldB, Ptuf butA. Figure S2. Growth curves and pH profiles of the 2,3-butanediol producer and parental strains, grown in 2×TY medium for 30 h at 160 rpm and 30 °C. Figure S3. 1H-NMR spectra of end-products of glucose metabolism in 2,3-butanediol producers. Figure S4. 1H-NMR spectra of end-products of glucose metabolism by wild type C. glutamicum under oxygen limiting conditions to illustrate the high reproducibility of the NMR measurements. Table S1. Growth parameters of producer strains as compared to the control strains. Table S2. Primers used in this study. Table S3. End-products of glucose metabolism and residual glucose measured in supernatants of cell suspensions of parent and producer strains incubated under oxygen limiting conditions (closed falcon tubes) for 48 h. Table S4. End-products of glucose metabolism and residual glucose measured in supernatants of cell suspensions of producer strains incubated under different aeration conditions for 30 h. Table S5. Summary of the best microbial 2,3-butanediol producers. [file 12934_2015_362_MOESM1_ESM.pptx]

## Slide 1
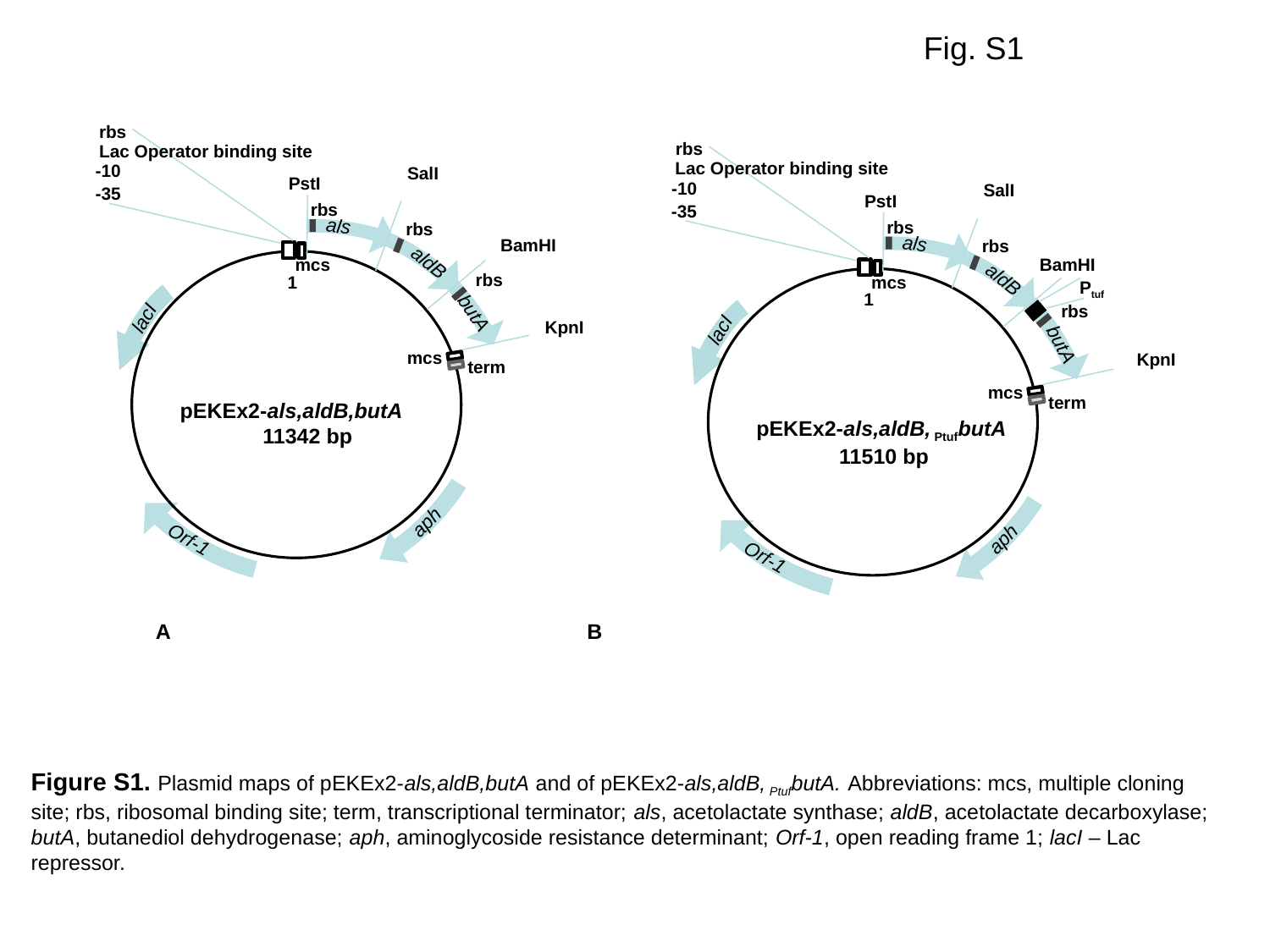

Fig. S1
rbs
rbs
Lac Operator binding site
Lac Operator binding site
-10
SalI
PstI
-10
SalI
-35
PstI
rbs
-35
lacI
rbs
rbs
lacI
als
als
BamHI
rbs
mcs
BamHI
rbs
1
mcs
aldB
Ptuf
1
rbs
KpnI
aldB
butA
mcs
KpnI
term
mcs
butA
term
pEKEx2-als,aldB,butA
11342 bp
pEKEx2-als,aldB, PtufbutA
11510 bp
aph
aph
Orf-1
Orf-1
A
B
Figure S1. Plasmid maps of pEKEx2-als,aldB,butA and of pEKEx2-als,aldB, PtufbutA. Abbreviations: mcs, multiple cloning site; rbs, ribosomal binding site; term, transcriptional terminator; als, acetolactate synthase; aldB, acetolactate decarboxylase; butA, butanediol dehydrogenase; aph, aminoglycoside resistance determinant; Orf-1, open reading frame 1; lacI – Lac repressor.

## Slide 2
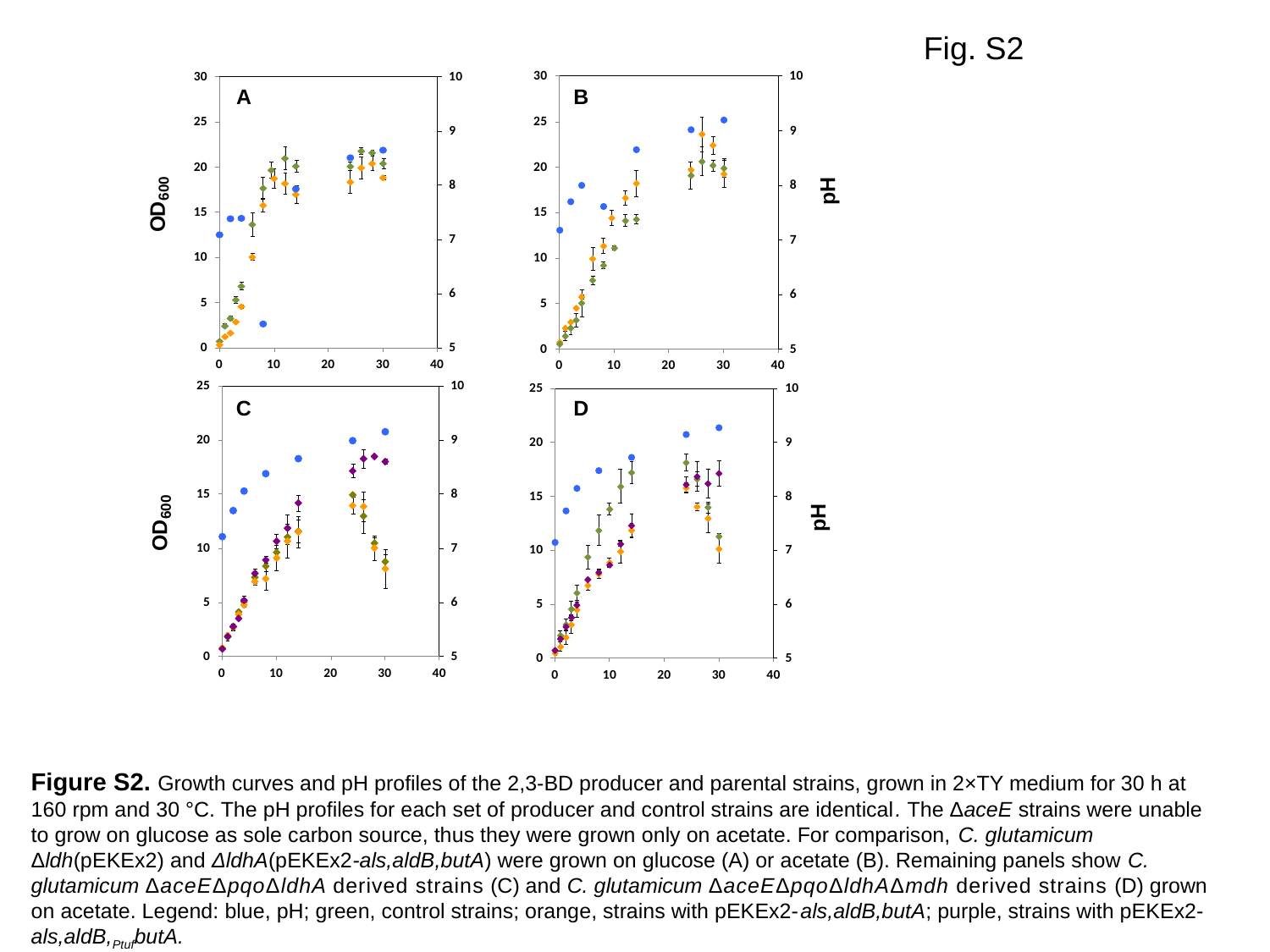

Fig. S2
Figure S2. Growth curves and pH profiles of the 2,3-BD producer and parental strains, grown in 2×TY medium for 30 h at 160 rpm and 30 °C. The pH profiles for each set of producer and control strains are identical. The ΔaceE strains were unable to grow on glucose as sole carbon source, thus they were grown only on acetate. For comparison, C. glutamicum Δldh(pEKEx2) and ΔldhA(pEKEx2-als,aldB,butA) were grown on glucose (A) or acetate (B). Remaining panels show C. glutamicum ΔaceEΔpqoΔldhA derived strains (C) and C. glutamicum ΔaceEΔpqoΔldhAΔmdh derived strains (D) grown on acetate. Legend: blue, pH; green, control strains; orange, strains with pEKEx2-als,aldB,butA; purple, strains with pEKEx2-als,aldB,PtufbutA.

## Slide 3
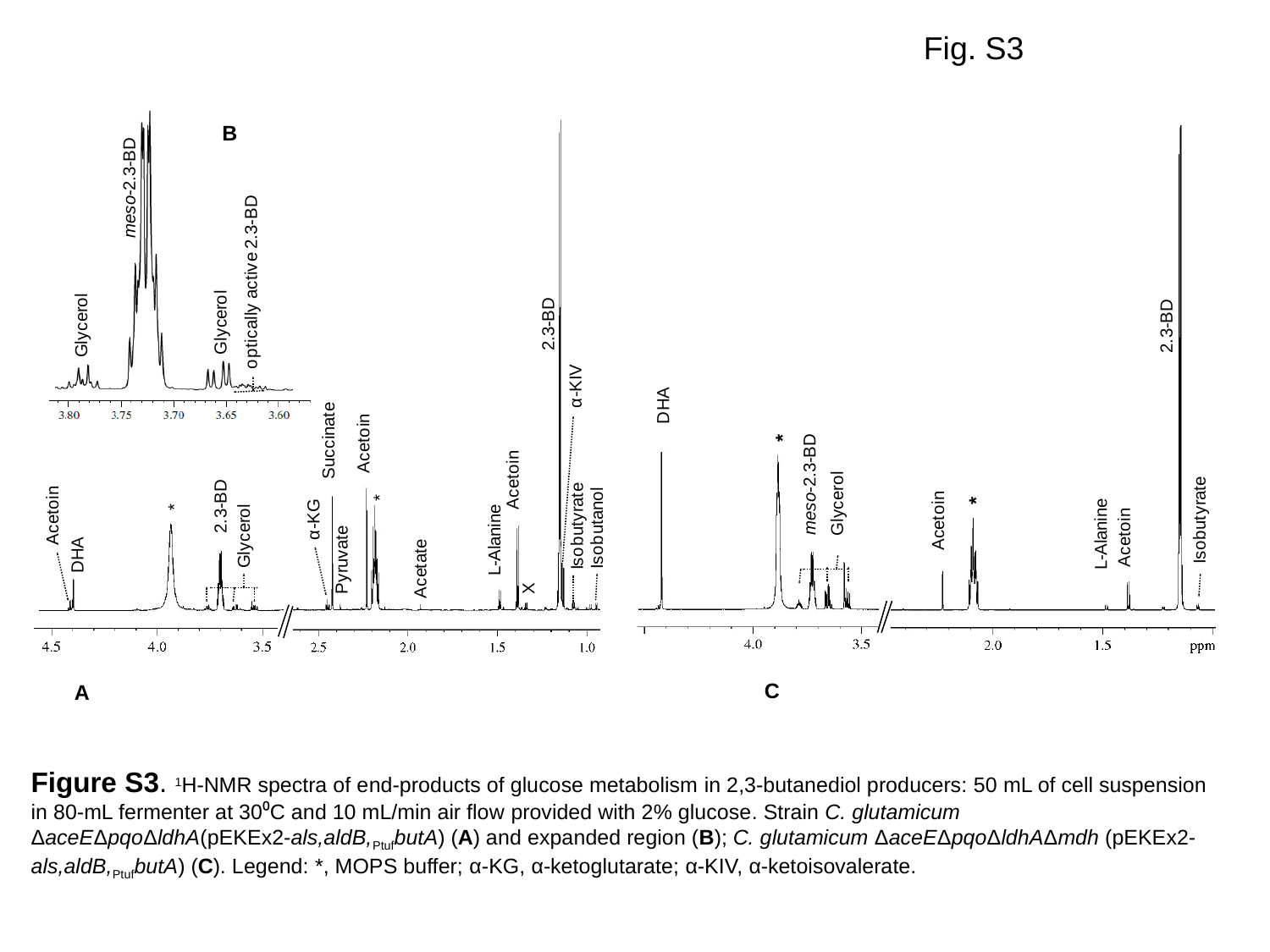

Fig. S3
Figure S3. 1H-NMR spectra of end-products of glucose metabolism in 2,3-butanediol producers: 50 mL of cell suspension in 80-mL fermenter at 30⁰C and 10 mL/min air flow provided with 2% glucose. Strain C. glutamicum ΔaceEΔpqoΔldhA(pEKEx2-als,aldB,PtufbutA) (A) and expanded region (B); C. glutamicum ΔaceEΔpqoΔldhAΔmdh (pEKEx2-als,aldB,PtufbutA) (C). Legend: *, MOPS buffer; α-KG, α-ketoglutarate; α-KIV, α-ketoisovalerate.

## Slide 4
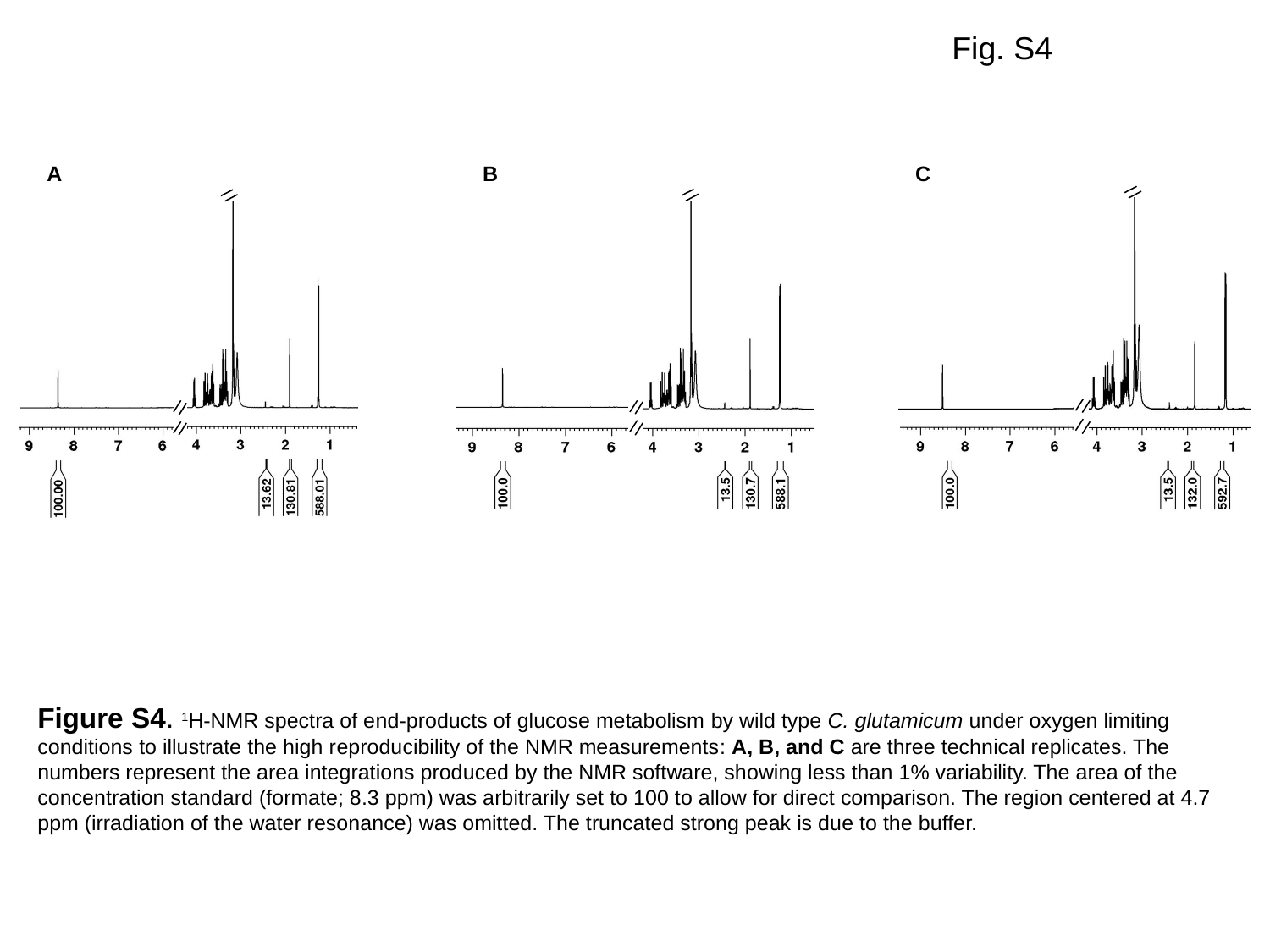

Fig. S4
A
B
C
Figure S4. 1H-NMR spectra of end-products of glucose metabolism by wild type C. glutamicum under oxygen limiting conditions to illustrate the high reproducibility of the NMR measurements: A, B, and C are three technical replicates. The numbers represent the area integrations produced by the NMR software, showing less than 1% variability. The area of the concentration standard (formate; 8.3 ppm) was arbitrarily set to 100 to allow for direct comparison. The region centered at 4.7 ppm (irradiation of the water resonance) was omitted. The truncated strong peak is due to the buffer.

## Slide 5
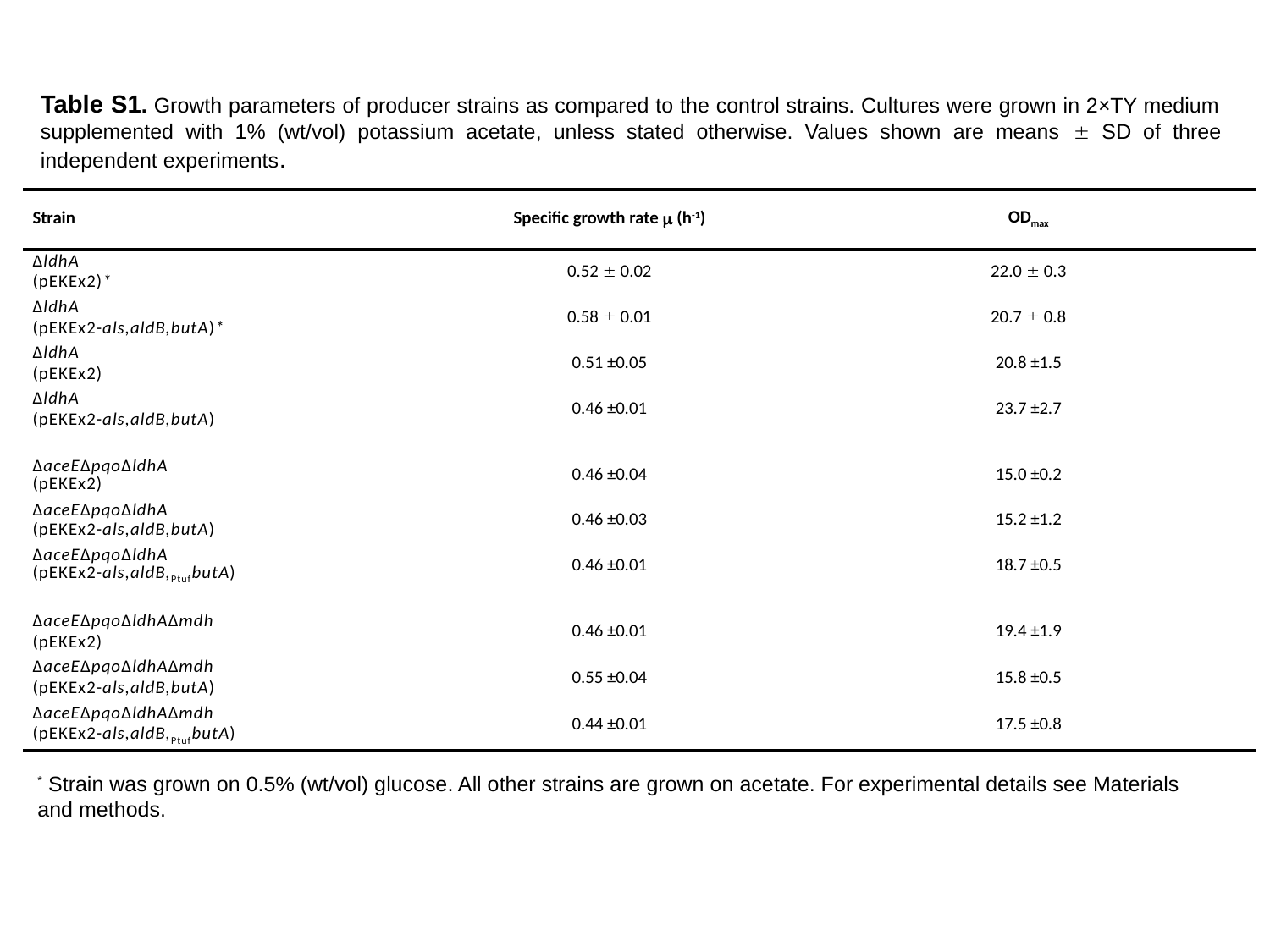

Table S1. Growth parameters of producer strains as compared to the control strains. Cultures were grown in 2×TY medium supplemented with 1% (wt/vol) potassium acetate, unless stated otherwise. Values shown are means ± SD of three independent experiments.
| Strain | Specific growth rate  (h-1) | ODmax |
| --- | --- | --- |
| ΔldhA (pEKEx2)\* | 0.52 ± 0.02 | 22.0 ± 0.3 |
| ΔldhA (pEKEx2-als,aldB,butA)\* | 0.58 ± 0.01 | 20.7 ± 0.8 |
| ΔldhA (pEKEx2) | 0.51 ±0.05 | 20.8 ±1.5 |
| ΔldhA (pEKEx2-als,aldB,butA) | 0.46 ±0.01 | 23.7 ±2.7 |
| | | |
| ΔaceEΔpqoΔldhA (pEKEx2) | 0.46 ±0.04 | 15.0 ±0.2 |
| ΔaceEΔpqoΔldhA (pEKEx2-als,aldB,butA) | 0.46 ±0.03 | 15.2 ±1.2 |
| ΔaceEΔpqoΔldhA (pEKEx2-als,aldB,PtufbutA) | 0.46 ±0.01 | 18.7 ±0.5 |
| | | |
| ΔaceEΔpqoΔldhAΔmdh (pEKEx2) | 0.46 ±0.01 | 19.4 ±1.9 |
| ΔaceEΔpqoΔldhAΔmdh (pEKEx2-als,aldB,butA) | 0.55 ±0.04 | 15.8 ±0.5 |
| ΔaceEΔpqoΔldhAΔmdh (pEKEx2-als,aldB,PtufbutA) | 0.44 ±0.01 | 17.5 ±0.8 |
* Strain was grown on 0.5% (wt/vol) glucose. All other strains are grown on acetate. For experimental details see Materials and methods.

## Slide 6
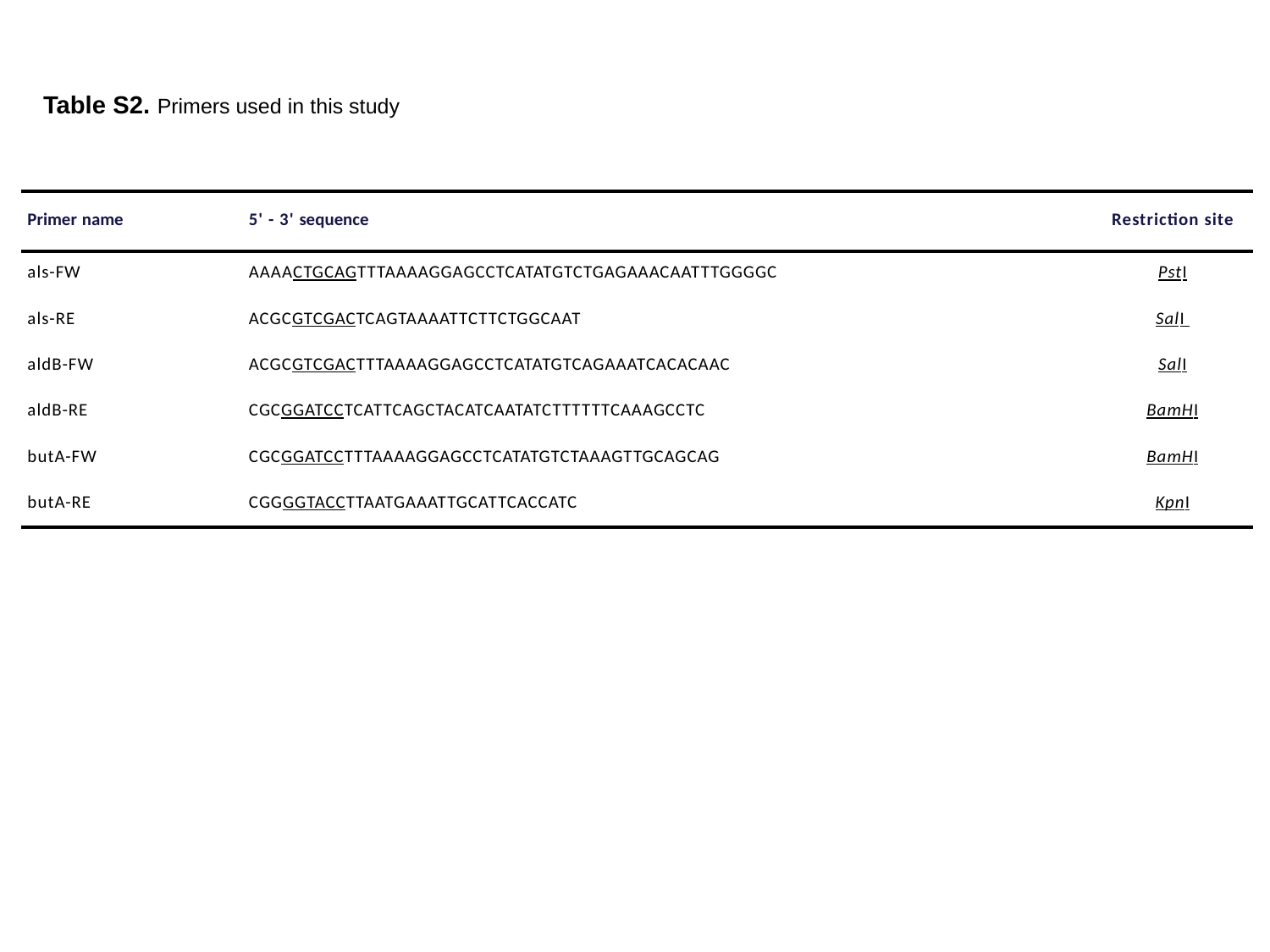

Table S2. Primers used in this study
| Primer name | 5' - 3' sequence | Restriction site |
| --- | --- | --- |
| als-FW | AAAACTGCAGTTTAAAAGGAGCCTCATATGTCTGAGAAACAATTTGGGGC | PstI |
| als-RE | ACGCGTCGACTCAGTAAAATTCTTCTGGCAAT | SalI |
| aldB-FW | ACGCGTCGACTTTAAAAGGAGCCTCATATGTCAGAAATCACACAAC | SalI |
| aldB-RE | CGCGGATCCTCATTCAGCTACATCAATATCTTTTTTCAAAGCCTC | BamHI |
| butA-FW | CGCGGATCCTTTAAAAGGAGCCTCATATGTCTAAAGTTGCAGCAG | BamHI |
| butA-RE | CGGGGTACCTTAATGAAATTGCATTCACCATC | KpnI |

## Slide 7
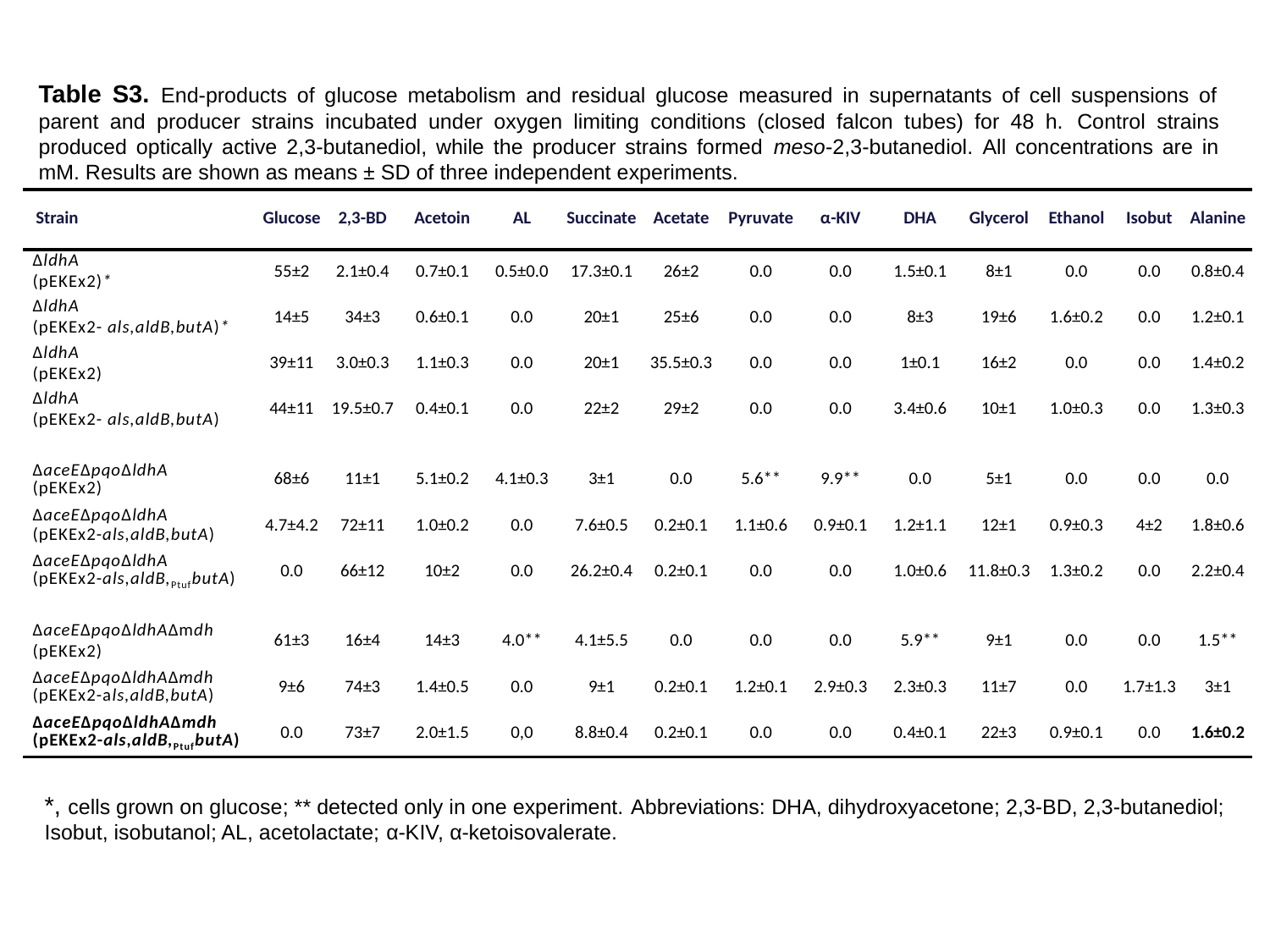

Table S3. End-products of glucose metabolism and residual glucose measured in supernatants of cell suspensions of parent and producer strains incubated under oxygen limiting conditions (closed falcon tubes) for 48 h. Control strains produced optically active 2,3-butanediol, while the producer strains formed meso-2,3-butanediol. All concentrations are in mM. Results are shown as means ± SD of three independent experiments.
| Strain | Glucose | 2,3-BD | Acetoin | AL | Succinate | Acetate | Pyruvate | α-KIV | DHA | Glycerol | Ethanol | Isobut | Alanine |
| --- | --- | --- | --- | --- | --- | --- | --- | --- | --- | --- | --- | --- | --- |
| ΔldhA (pEKEx2)\* | 55±2 | 2.1±0.4 | 0.7±0.1 | 0.5±0.0 | 17.3±0.1 | 26±2 | 0.0 | 0.0 | 1.5±0.1 | 8±1 | 0.0 | 0.0 | 0.8±0.4 |
| ΔldhA (pEKEx2- als,aldB,butA)\* | 14±5 | 34±3 | 0.6±0.1 | 0.0 | 20±1 | 25±6 | 0.0 | 0.0 | 8±3 | 19±6 | 1.6±0.2 | 0.0 | 1.2±0.1 |
| ΔldhA (pEKEx2) | 39±11 | 3.0±0.3 | 1.1±0.3 | 0.0 | 20±1 | 35.5±0.3 | 0.0 | 0.0 | 1±0.1 | 16±2 | 0.0 | 0.0 | 1.4±0.2 |
| ΔldhA (pEKEx2- als,aldB,butA) | 44±11 | 19.5±0.7 | 0.4±0.1 | 0.0 | 22±2 | 29±2 | 0.0 | 0.0 | 3.4±0.6 | 10±1 | 1.0±0.3 | 0.0 | 1.3±0.3 |
| | | | | | | | | | | | | | |
| ΔaceEΔpqoΔldhA (pEKEx2) | 68±6 | 11±1 | 5.1±0.2 | 4.1±0.3 | 3±1 | 0.0 | 5.6\*\* | 9.9\*\* | 0.0 | 5±1 | 0.0 | 0.0 | 0.0 |
| ΔaceEΔpqoΔldhA (pEKEx2-als,aldB,butA) | 4.7±4.2 | 72±11 | 1.0±0.2 | 0.0 | 7.6±0.5 | 0.2±0.1 | 1.1±0.6 | 0.9±0.1 | 1.2±1.1 | 12±1 | 0.9±0.3 | 4±2 | 1.8±0.6 |
| ΔaceEΔpqoΔldhA (pEKEx2-als,aldB,PtufbutA) | 0.0 | 66±12 | 10±2 | 0.0 | 26.2±0.4 | 0.2±0.1 | 0.0 | 0.0 | 1.0±0.6 | 11.8±0.3 | 1.3±0.2 | 0.0 | 2.2±0.4 |
| | | | | | | | | | | | | | |
| ΔaceEΔpqoΔldhAΔmdh (pEKEx2) | 61±3 | 16±4 | 14±3 | 4.0\*\* | 4.1±5.5 | 0.0 | 0.0 | 0.0 | 5.9\*\* | 9±1 | 0.0 | 0.0 | 1.5\*\* |
| ΔaceEΔpqoΔldhAΔmdh (pEKEx2-als,aldB,butA) | 9±6 | 74±3 | 1.4±0.5 | 0.0 | 9±1 | 0.2±0.1 | 1.2±0.1 | 2.9±0.3 | 2.3±0.3 | 11±7 | 0.0 | 1.7±1.3 | 3±1 |
| ΔaceEΔpqoΔldhAΔmdh (pEKEx2-als,aldB,PtufbutA) | 0.0 | 73±7 | 2.0±1.5 | 0,0 | 8.8±0.4 | 0.2±0.1 | 0.0 | 0.0 | 0.4±0.1 | 22±3 | 0.9±0.1 | 0.0 | 1.6±0.2 |
*, cells grown on glucose; ** detected only in one experiment. Abbreviations: DHA, dihydroxyacetone; 2,3-BD, 2,3-butanediol; Isobut, isobutanol; AL, acetolactate; α-KIV, α-ketoisovalerate.

## Slide 8
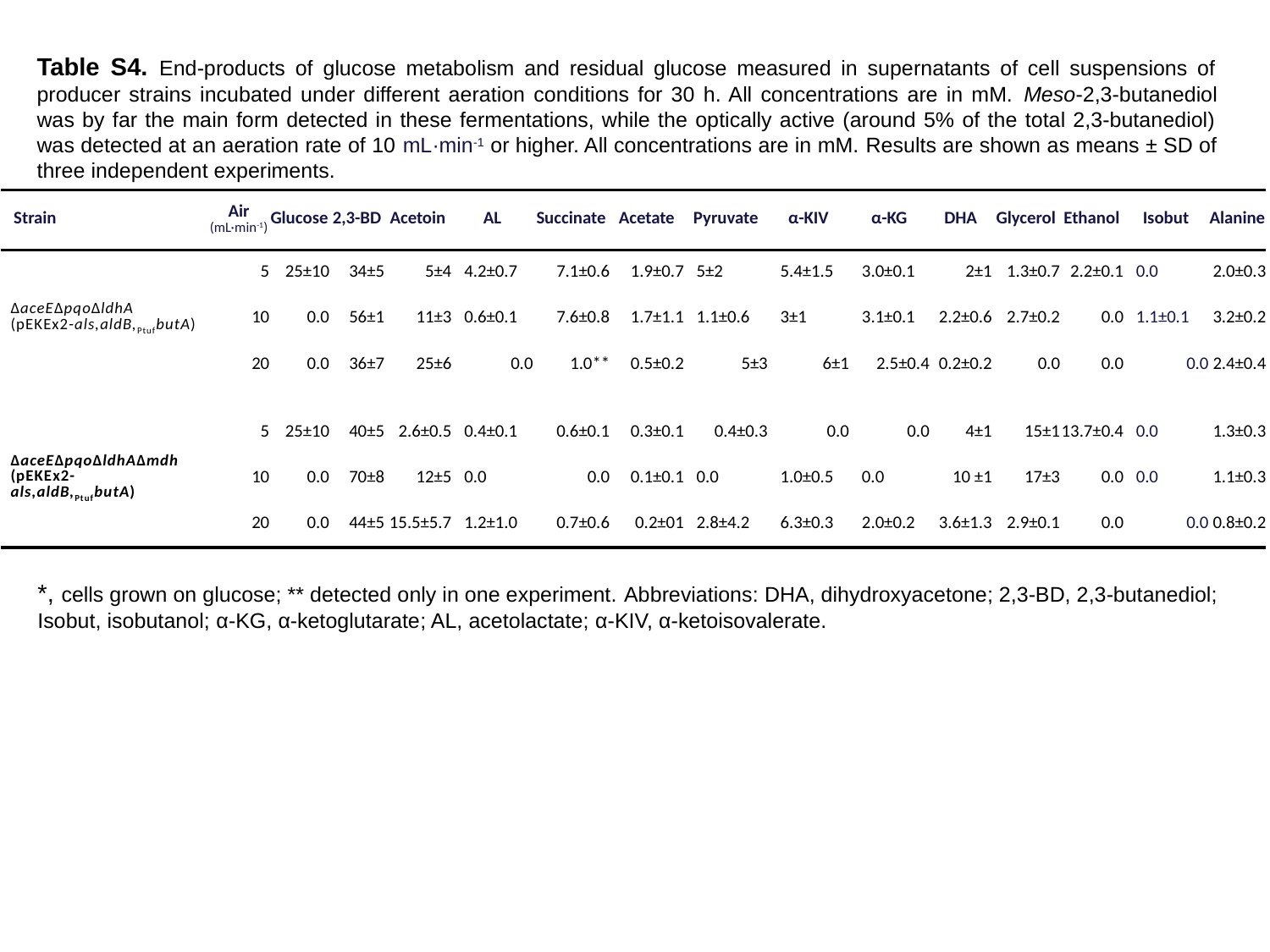

Table S4. End-products of glucose metabolism and residual glucose measured in supernatants of cell suspensions of producer strains incubated under different aeration conditions for 30 h. All concentrations are in mM. Meso-2,3-butanediol was by far the main form detected in these fermentations, while the optically active (around 5% of the total 2,3-butanediol) was detected at an aeration rate of 10 mL·min-1 or higher. All concentrations are in mM. Results are shown as means ± SD of three independent experiments.
| Strain | Air (mL·min-1) | Glucose | 2,3-BD | Acetoin | AL | Succinate | Acetate | Pyruvate | α-KIV | α-KG | DHA | Glycerol | Ethanol | Isobut | Alanine |
| --- | --- | --- | --- | --- | --- | --- | --- | --- | --- | --- | --- | --- | --- | --- | --- |
| ΔaceEΔpqoΔldhA (pEKEx2-als,aldB,PtufbutA) | 5 | 25±10 | 34±5 | 5±4 | 4.2±0.7 | 7.1±0.6 | 1.9±0.7 | 5±2 | 5.4±1.5 | 3.0±0.1 | 2±1 | 1.3±0.7 | 2.2±0.1 | 0.0 | 2.0±0.3 |
| | 10 | 0.0 | 56±1 | 11±3 | 0.6±0.1 | 7.6±0.8 | 1.7±1.1 | 1.1±0.6 | 3±1 | 3.1±0.1 | 2.2±0.6 | 2.7±0.2 | 0.0 | 1.1±0.1 | 3.2±0.2 |
| | 20 | 0.0 | 36±7 | 25±6 | 0.0 | 1.0\*\* | 0.5±0.2 | 5±3 | 6±1 | 2.5±0.4 | 0.2±0.2 | 0.0 | 0.0 | 0.0 | 2.4±0.4 |
| | | | | | | | | | | | | | | | |
| ΔaceEΔpqoΔldhAΔmdh (pEKEx2-als,aldB,PtufbutA) | 5 | 25±10 | 40±5 | 2.6±0.5 | 0.4±0.1 | 0.6±0.1 | 0.3±0.1 | 0.4±0.3 | 0.0 | 0.0 | 4±1 | 15±1 | 13.7±0.4 | 0.0 | 1.3±0.3 |
| | 10 | 0.0 | 70±8 | 12±5 | 0.0 | 0.0 | 0.1±0.1 | 0.0 | 1.0±0.5 | 0.0 | 10 ±1 | 17±3 | 0.0 | 0.0 | 1.1±0.3 |
| | 20 | 0.0 | 44±5 | 15.5±5.7 | 1.2±1.0 | 0.7±0.6 | 0.2±01 | 2.8±4.2 | 6.3±0.3 | 2.0±0.2 | 3.6±1.3 | 2.9±0.1 | 0.0 | 0.0 | 0.8±0.2 |
*, cells grown on glucose; ** detected only in one experiment. Abbreviations: DHA, dihydroxyacetone; 2,3-BD, 2,3-butanediol; Isobut, isobutanol; α-KG, α-ketoglutarate; AL, acetolactate; α-KIV, α-ketoisovalerate.

## Slide 9
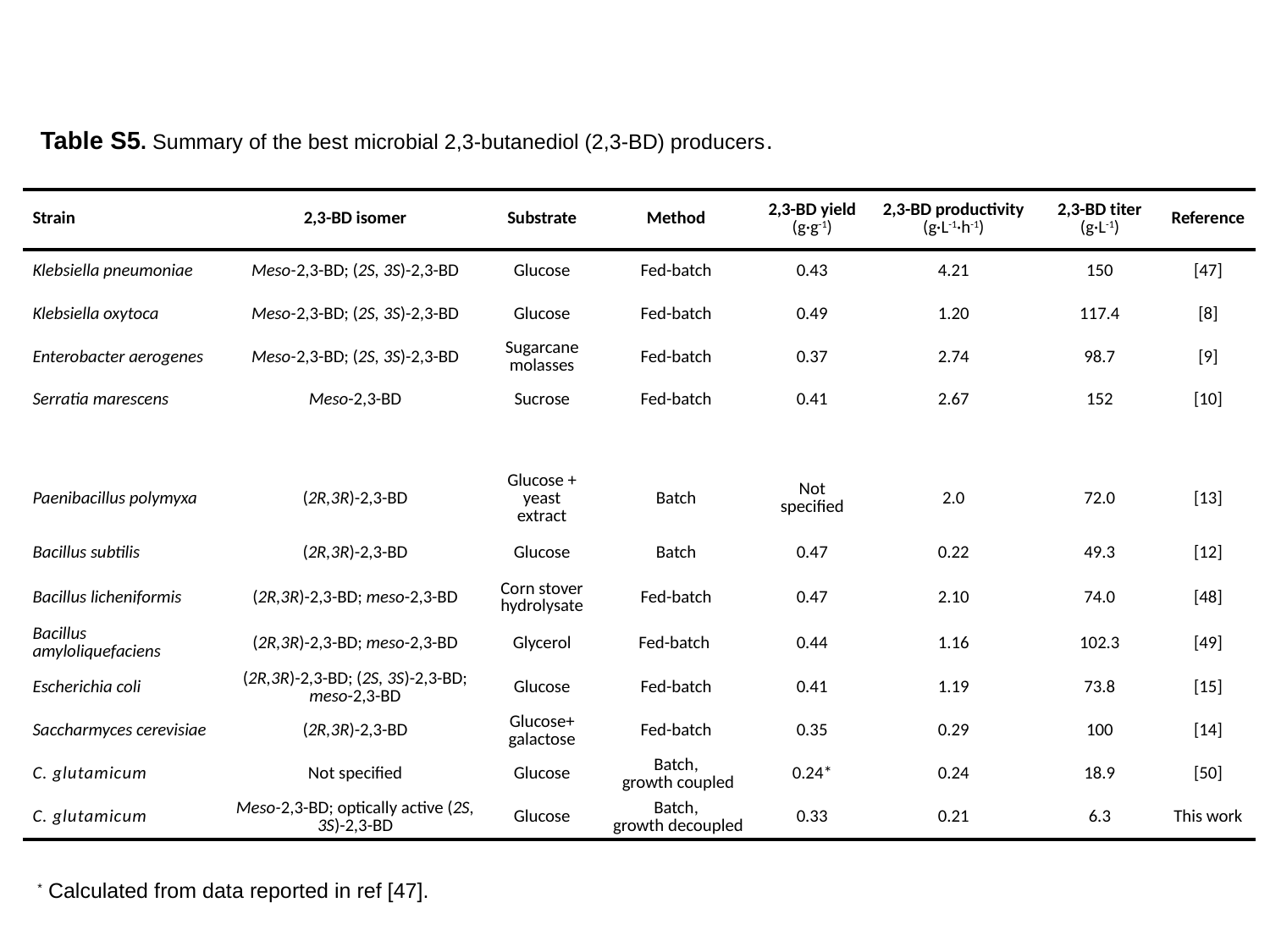

Table S5. Summary of the best microbial 2,3-butanediol (2,3-BD) producers.
| Strain | 2,3-BD isomer | Substrate | Method | 2,3-BD yield (g·g-1) | 2,3-BD productivity (g·L-1·h-1) | 2,3-BD titer (g·L-1) | Reference |
| --- | --- | --- | --- | --- | --- | --- | --- |
| Klebsiella pneumoniae | Meso-2,3-BD; (2S, 3S)-2,3-BD | Glucose | Fed-batch | 0.43 | 4.21 | 150 | [47] |
| Klebsiella oxytoca | Meso-2,3-BD; (2S, 3S)-2,3-BD | Glucose | Fed-batch | 0.49 | 1.20 | 117.4 | [8] |
| Enterobacter aerogenes | Meso-2,3-BD; (2S, 3S)-2,3-BD | Sugarcane molasses | Fed-batch | 0.37 | 2.74 | 98.7 | [9] |
| Serratia marescens | Meso-2,3-BD | Sucrose | Fed-batch | 0.41 | 2.67 | 152 | [10] |
| | | | | | | | |
| Paenibacillus polymyxa | (2R,3R)-2,3-BD | Glucose + yeast extract | Batch | Not specified | 2.0 | 72.0 | [13] |
| Bacillus subtilis | (2R,3R)-2,3-BD | Glucose | Batch | 0.47 | 0.22 | 49.3 | [12] |
| Bacillus licheniformis | (2R,3R)-2,3-BD; meso-2,3-BD | Corn stover hydrolysate | Fed-batch | 0.47 | 2.10 | 74.0 | [48] |
| Bacillus amyloliquefaciens | (2R,3R)-2,3-BD; meso-2,3-BD | Glycerol | Fed-batch | 0.44 | 1.16 | 102.3 | [49] |
| Escherichia coli | (2R,3R)-2,3-BD; (2S, 3S)-2,3-BD; meso-2,3-BD | Glucose | Fed-batch | 0.41 | 1.19 | 73.8 | [15] |
| Saccharmyces cerevisiae | (2R,3R)-2,3-BD | Glucose+ galactose | Fed-batch | 0.35 | 0.29 | 100 | [14] |
| C. glutamicum | Not specified | Glucose | Batch, growth coupled | 0.24\* | 0.24 | 18.9 | [50] |
| C. glutamicum | Meso-2,3-BD; optically active (2S, 3S)-2,3-BD | Glucose | Batch, growth decoupled | 0.33 | 0.21 | 6.3 | This work |
* Calculated from data reported in ref [47].
